# Consolidative nivolumab versus observation in unresectable stage III non-small cell lung cancer patients following neoadjuvant nivolumab plus chemotherapy and concurrent chemoradiotherapy (CA209-7AL): a randomized clinical trial

**DOI:** 10.1038/s41392-025-02408-3

**Published:** 2025-09-29

**Authors:** Bo Qiu, Yuanyuan Zhao, Wenzhuo He, Weijin Zeng, Hongmei Zhang, Weineng Feng, Jun Jia, Daodu Wang, Daquan Wang, Fangjie Liu, Songran Liu, Shaohan Yin, Chuanmiao Xie, Rui Zhou, Yi Hu, Qianwen Liu, Jinyu Guo, Suping Guo, Yingjia Wu, Qiaoting Luo, Jibin Li, Yunpeng Yang, Liangping Xia, Li Zhang, Hui Liu

**Affiliations:** 1 Department of Radiation Oncology, State Key Laboratory of Oncology in South China, Collaborative Innovation Center for Cancer Medicine, Sun Yat-sen University Cancer Center, Guangzhou, China; 2 Department of Medical Oncology, State Key Laboratory of Oncology in South China, Collaborative Innovation Center for Cancer Medicine, Sun Yat-sen University Cancer Center, Guangzhou, China; 3 Department of VIP Region, State Key Laboratory of Oncology in South China, Collaborative Innovation Center for Cancer Medicine, Sun Yat-sen University Cancer Center, Guangzhou, China; 4 Department of Radiation Oncology, Shanwei Yihui Fund Hospital, Shanwei, China; 5 Department of Oncology, Air Force Hospital of Southern Theater Command of the People’s Liberation Army, Guangdong, China; 6 Department of Pulmonary Oncology, The First People’s Hospital of Foshan, Foshan, China; 7 Department of Medical Oncology, Affiliated Dongguan Hospital, Southern Medical University, Dongguan, China; 8 Department of Surgical Oncology, Shanwei Yihui Fund Hospital, Shanwei, China; 9 Department of Pathology, State Key Laboratory of Oncology in South China, Collaborative Innovation Center for Cancer Medicine, Sun Yat-sen University Cancer Center, Guangzhou, China; 10 Department of Radiology, State Key Laboratory of Oncology in South China, Collaborative Innovation Center for Cancer Medicine, Sun Yat-sen University Cancer Center, Guangzhou, China; 11 Department of Thoracic Surgery, State Key Laboratory of Oncology in South China, Collaborative Innovation Center for Cancer Medicine, Sun Yat-sen University Cancer Center, Guangzhou, China; 12 Department of Clinical Research, State Key Laboratory of Oncology in South China, Collaborative Innovation Center for Cancer Medicine, Sun Yat-sen University Cancer Center, Guangzhou, China

**Keywords:** Lung cancer, Clinical trials

## Abstract

CA209-7AL is a randomized, multicenter, phase 2 trial evaluating the efficacy and safety of consolidative nivolumab (NIVO) versus observation following neoadjuvant NIVO plus chemotherapy and concurrent chemoradiotherapy (CCRT) for unresectable stage III NSCLC. Patients received 2 cycles of neoadjuvant chemo-NIVO therapy (docetaxel + cisplatin + NIVO) and CCRT (total dose 54–64 Gy). Post-CCRT, eligible patients were randomized 1:1 to receive consolidative NIVO (360 mg every 3 weeks for up to 12 months) or observation. The primary endpoint was progression-free survival (PFS) from randomization. Between December 3rd, 2019, and August 18th, 2023, 264 patients were enrolled, and 172 were randomized to NIVO consolidation (*n* = 86) or observation (*n* = 86). With a median follow-up of 22·8 months, NIVO consolidation resulted in significantly longer PFS than did observation (median not reached vs. 12.2 months [95% CI 10.2–20.8]; stratified hazard ratio 0·49 [95% CI 0.30–0.79], *p* = 0.003). NIVO consolidation also demonstrated superior PFS compared with a parallel real-world study, where patients received CCRT followed by consolidative immunotherapy (median PFS: 15.7 months [95% CI 11.9-NA]). Grade 3 or 4 toxicities occurred in 9.3% of patients in the consolidation group versus 4·6% in the observation group, with similar rates of pneumonitis (2.3% each) and proximal bronchial tree toxicity (3.5% vs. 2.3%). Treatment-related death occurred in 1 (1.2%) patient in the consolidation group because of pneumonitis. Patients with a high TMB had a longer PFS with consolidation (NR vs. 15.2 months, *p* = 0.042). Consolidative NIVO following neoadjuvant NIVO plus chemotherapy and CCRT demonstrated effectiveness and tolerability for patients with unresectable stage III NSCLC (ClinicalTrials.gov NCT04085250).

## Introduction

Lung cancer is the leading cause of cancer death worldwide, with non-small cell lung cancer (NSCLC) representing approximately 80–85% of all cases.^1^ Neoadjuvant nivolumab (NIVO) plus chemotherapy has shown promising response rates, survival outcomes and safety profiles, establishing it as the standard treatment for eligible patients with resectable NSCLC, including those with stage III disease.^2–4^ In the phase III CheckMate 816 trial for resectable stage IB to IIIA NSCLC, the combination of neoadjuvant chemotherapy and NIVO achieved an overall response rate (ORR) of 53.6% and a pathological complete response rate of 24%, along with a significant survival benefit (median event-free survival [EFS] of 31.6 vs. 20.8 months, hazard ratio [HR] 0.63, *P* = 0.005).^4^ Moreover, a perioperative approach, which includes neoadjuvant therapy followed by surgery and adjuvant therapy, with immunotherapy-based treatments has demonstrated survival benefits.^5–7^ The phase III CheckMate 77 T study revealed that neoadjuvant NIVO plus chemotherapy followed by surgery and adjuvant NIVO significantly improved EFS compared with chemotherapy alone in patients with resectable IIA to IIIB NSCLC (HR 0.58, *P* < 0.001).^5^

Patients with unresectable stage III NSCLC exhibit a heightened need for neoadjuvant chemoimmunotherapy due to larger tumor volumes and an elevated risk of metastasis. Administering neoadjuvant NIVO in combination with chemotherapy prior to concurrent chemoradiotherapy (CCRT) offers several potential benefits for these patients. First, although consolidative immunotherapy following CCRT has been shown to significantly prolong progression-free survival (PFS) and overall survival (OS),^8,9^ many patients cannot reach the consolidative phase due to toxicity or early disease progression post-CCRT, particularly those with bulky tumors or stage IIIB-C disease.^10,11^ Neoadjuvant chemoimmunotherapy can potentially reduce the tumor burden, thereby increasing the efficacy of CCRT and reducing radiation-associated toxicity,^12,13^ enabling more patients to qualify for consolidative immunotherapy post-CCRT. Second, administering immunotherapy in the neoadjuvant setting, which involves a less compromised immune system and intact tumors, may stimulate more robust activation of antitumor T cells, potentially improving clinical outcomes compared with postradical treatment administration.^14^ Despite these potential advantages, research on neoadjuvant immunotherapy plus chemotherapy prior to CCRT for stage III NSCLC remains limited. A retrospective study explored the feasibility of neoadjuvant chemoimmunotherapy before CCRT for unresectable stage III NSCLC and reported a 12-month PFS rate of 85.8% (95% CI, 78.0–94.4%).^15^ These findings underscore the potential viability of neoadjuvant chemoimmunotherapy as a therapeutic strategy for patients with unresectable stage III NSCLC. However, it remains uncertain whether consolidative immunotherapy following neoadjuvant chemoimmunotherapy and CCRT can further increase the treatment efficacy in patients with unresectable stage III NSCLC. Furthermore, it is unclear whether this treatment strategy provides better outcomes than those reported in the PACIFIC trial.

Here, we report the efficacy and safety results from the prespecified interim analysis of the phase 2, randomized CA209-7AL trial, which evaluated consolidative NIVO versus observation following neoadjuvant NIVO plus chemotherapy and CCRT in patients with unresectable stage III NSCLC. We concurrently report the clinical outcomes of a parallel real-world study (RWS), which included patients with unresectable stage III NSCLC who declined to participate in the CA209-7AL trial and received standard-of-care CCRT followed by consolidative immunotherapy.

## Results

### Patients

**Fig. 1:**
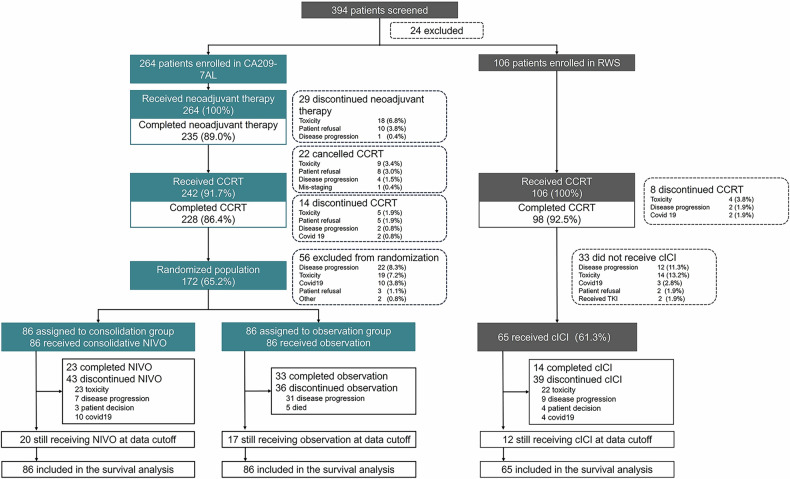
Study profile and treatment disposition. The data cutoff date was January 31, 2024. CCRT concurrent chemoradiotherapy, NIVO nivolumab, cICI consolidative immunotherapy, RWS real-world study

Between Dec 3rd, 2019, and Aug 18th, 2023, a total of 264 patients were enrolled in CA209-7AL to receive neoadjuvant chemo-NIVO therapy, 242 of whom received CCRT, and 172 patients were randomly assigned to NIVO consolidation (*n* = 86) or to observation (*n* = 86) after CCRT (Fig. 1). The patient demographics and disease characteristics of the enrolled patients (*n* = 264) are listed in Table 1. The median age of all patients was 60 years (IQR 30–75), and the majority were men (218 [82.6%]) or smokers (172 [65.2%]). Approximately half of the patients had an Eastern Cooperative Oncology Group (ECOG) performance status of 1 (124 [47.0%]), and more than half of the patients had squamous cell carcinoma (136 [51.5%]). There were 127 (48.1%) patients with stage IIIB disease and 87 (33.0%) with stage IIIC disease. EGFR mutations were observed in 22 patients (8.3%). PD-L1 expression of 1% or more on tumor cells occurred in 76 (28.8%) patients; PD-L1 expression of less than 1% occurred in 58 (22.0%) patients; and PD-L1 status was unknown in 130 (49.2%) patients. The randomly assigned patients (*n* = 172) had similar clinical characteristics. The baseline characteristics were well balanced between the consolidation and observation groups.

**Table 1:** Baseline characteristics of patients in CA209-7AL (*N* = 264) and the real-world study (*N* = 106)

	CA 209-7AL				RWS	
	All (*N* = 264)	Randomized patients (*N* = 172)	Consolidation group (*N* = 86)	Observation group (*N* = 86)	All (*N* = 106)	cICI cohort (*N* = 65)
Age, years	60 (30–75)	59 (30–75)	57 (39–73)	62(30–75)	59 (39–75)	58 (39–75)
<65	171 (64.8%)	112 (65.1%)	56 (65.1%)	56 (65.1%)	75 (70.8%)	47 (72.3%)
≥65	93 (35.2%)	60 (34.9%)	30 (34.9)	30 (34.9)	31 (29.2%)	18 (27.7%)
Sex						
Male	218 (82.6%)	141 (82.0%)	71 (82.6%)	70 (81.4%)	88 (83.0%)	54 (83.1%)
Female	46 (17.4%)	31 (18.0%)	15 (17.4%)	16 (18.6%)	18 (17.0%)	11 (16.9%)
ECOG PS						
0	140 (53.0%)	112 (65.1%)	57 (66.3%)	55 (64.0%)	59 (55.7%)	40 (61.5%)
1	124 (47.0%)	60 (34.9%)	29 (33.7%)	31 ((36.0%)	47 (44.3%)	25 (38.5%)
Smoking history						
Non-smoker	92 (34.8%)	57 (33.1%)	28 (32.6%)	29 (33.7%)	33 (31.1%)	23 (35.4%)
Smoker	172 (65.2%)	115 (66.9%)	58 (67.4%)	57 (66.3%)	73 (68.9%)	42 (64.6%)
UICC/AJCC Stage (8th)						
IIIA	50 (18.9%)	37 (21.5%)	17 (19.8%)	20 (23.3%)	24 (22.6%)	16 (24.6%)
IIIB	127(48.1%)	86 (50.0%)	46 (53.5%)	40 (46.5%)	54 (50.9%)	30 (46.2%)
IIIC	87 (33.0%)	49 (28.5%)	23 (26.7%)	26 (30.2%)	28 (26.4%)	19 (29.2%)
Histology						
Squamous	136 (51.5%)	83 (48.3%)	38 (44.2%)	45 (52.3%)	59 (55.7%)	37 (56.9%)
Non-squamous	113 (42.8%)	80 (46.5%)	43 (50.0%)	37 (43.0%)	45 (42.5%)	26 (40.0%)
NOS	15 (5.7%)	9 (5.2%)	5 (5.8%)	4 (4.7%)	2 (1.9%)	2 (3.1%)
EGFR status						
Wild	242 (91.7%)	157 (91.3%)	78 (90.7%)	79 (91.9%)	99 (93.4%)	61 (93.8%)
Mutant	22 (8.3%)	15 (8.7%)	8 (9.3%)	7 (8.1%)	7 (6.6%)	4 (6.2%)
PD-L1 expression^a^						
<1%	58 (22.0%)	43 (25.0%)	23 (26.7%)	20 (23.3%)	20 (18.9%)	14 (21.5%)
≥1%	76 (28.8%)	68 (39.5%)	38 (44.2%)	30 (34.9%)	35 (33.0%)	26 (40.0%)
Missing	130 (49.2%)	61 (35.5%)	25 (29.1%)	36 (41.9%)	51 (48.1%)	25 (38.5%)

Data are median (range) or *n* (%). Assessment of baseline PD-L1 expression was not mandatory for study enrollment

*cICI* consolidative immunotherapy, *ECOG PS* Eastern Cooperative Oncology Group performance status, *EGFR* epidermal growth factor receptor, *NOS* Not Otherwise Specified, *RWS* real-world study

^a^ Determined by the PD-L1 IHC C22C3 pharmDx assay

A total of 106 patients who declined to participate in CA209-7AL were enrolled in the RWS, where they received CCRT followed by consolidative immunotherapy in clinical practice (Fig. 1). The baseline characteristics of patients in the RWS cohort were comparable to those in the CA209-7AL cohort (Table 1).

### Neoadjuvant chemo-NIVO therapy summary in CA209-7AL patients

All the enrolled 264 patients received at least 1 cycle of neoadjuvant chemo-NIVO therapy. Among the 264 patients, 235 (89.0%) completed prespecified neoadjuvant chemo-NIVO therapy. The reasons for discontinuing neoadjuvant chemo-NIVO therapy are listed in Fig. 1.

**Fig. 2:**
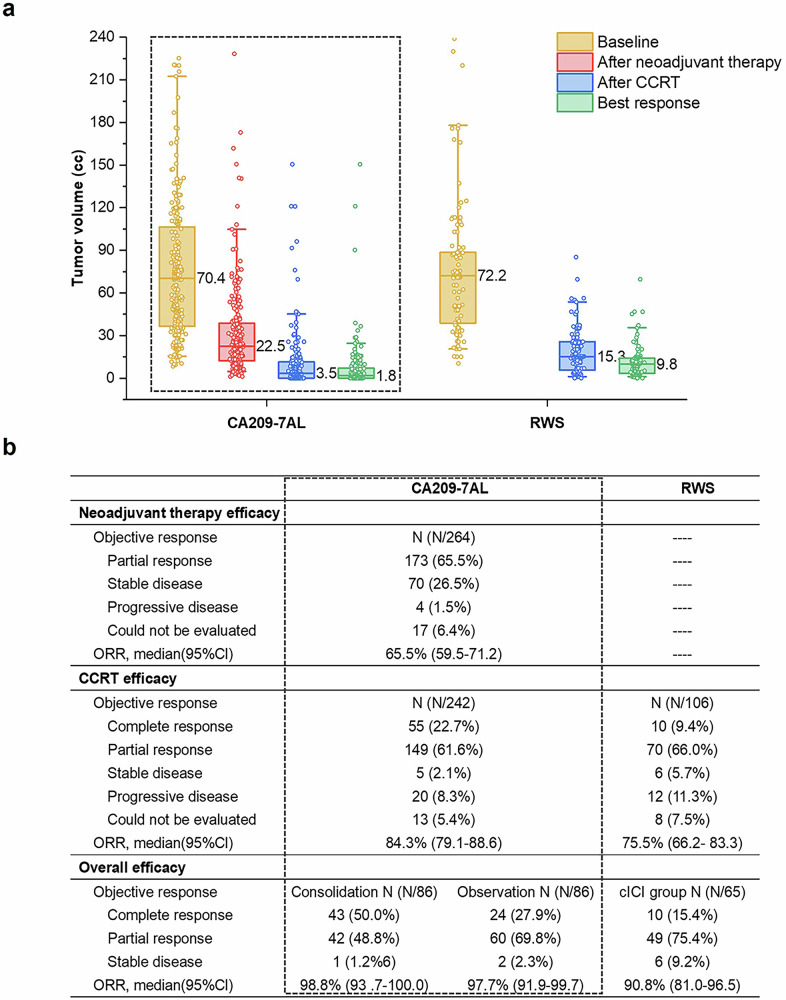
Antitumor activity. **a** Depicts the change in tumor volume during treatment in the CA209-7AL and RWS cohorts. The boxes represent the interquartile ranges of the tumor volumes. Whiskers depict the 5–95% percentiles of the tumor volumes. Lines represent the medians of the tumor volumes. Dots represent the individual data. **b** Shows the objective responses to neoadjuvant therapy, CCRT and the best overall responses. CCRT concurrent chemoradiation, RWS real-world study

Two weeks after neoadjuvant chemo-NIVO therapy, 173 (65.5%), 70 (26.5%) and 4 (1.5%) of the 264 patients experienced partial remission (PR), stable disease (SD), and progressive disease (PD), respectively (response data were missing for the other 17 [6.4%] patients). The objective response rate (ORR) to neoadjuvant chemo-NIVO therapy was 65.5% (95% CI 59.5–71.2%). The median tumor volume decreased from 70.4 cc (IQR 36.5–106.5) at baseline to 22.5 cc (IQR 12.4–38.6) after neoadjuvant chemo-NIVO therapy (Fig. 2). Together, the ORR and tumor volume reduction demonstrate that neoadjuvant chemo-NIVO significantly reduced tumor burden.

### CCRT summary in CA209-7AL

A total of 242 patients received CCRT, and 228 (228/242, 94.2%) patients completed CCRT per protocol. The reasons for canceling or discontinuing CCRT are listed in Fig. 1. Details about CCRT are provided in supplementary Table 1.

Two months after CCRT, 55 (22.7%), 149 (61.6%), 5 (2.1%) and 20 (8.3%) of 242 patients achieved complete remission (CR), PR, SD, and PD, respectively. Response data were missing for the other 13 (5.4%) patients. The ORR after CCRT was 84.3% (95% CI 79.1–88.6%) (Fig. 2). The most common site of disease progression after CCRT was the brain (13/20, 65.0%).

### Nivolumab consolidation or observation in CA209-7AL patients

A total of 172 patients who had completed neoadjuvant chemo-NIVO therapy and CCRT were randomly assigned to NIVO consolidation (*n* = 86) or observation (*n* = 86) groups. The reasons for exclusion from randomization following CCRT included disease progression (22/264, 8.3%), toxicity (19/264, 7.2%), acute COVID-19 infection (10/264, 3.8%), patient refusal (3/264, 1.1%) and others (2/264, 0.8%).

As of the data cutoff date for the interim analysis (January 31, 2024), the median duration of follow-up was 22.8 months (range, 0·7–43.5) for all randomized patients. Among the 86 patients in the consolidation group, 23 completed consolidation therapy; 20 were still receiving NIVO; and 43 patients discontinued NIVO due to disease progression (*n* = 7), toxicity (*n* = 23), patients’ decision (*n* = 3) or COVID-19 (*n* = 10). Toxicities were the primary reason for the discontinuation of consolidative NIVO. Among these, immune-related adverse events, including pneumonitis (*n* = 10), musculoskeletal pain (*n* = 2), and colitis (*n* = 1), were the most common causes (*n* = 13). Other toxicities leading to discontinuation included radiation-related proximal bronchial tree toxicity (*n* = 6), radiation pneumonitis (*n* = 3) and pneumonia (*n* = 1). For patients who completed or discontinued NIVO, the median number of cycles of consolidation infusions was 8 (IQR 3–13).

The best overall response of the 172 randomized patients is presented in Fig. 2. In the consolidation group, 43 (50.0%), 42 (48.8%) and 1 (1.2%) of 86 patients achieved CR, PR and SD, respectively, as their best overall response. The ORR was 98.8% (95% CI 93.7–100.0). In the observation group, 24 (27.9%), 60 (69.8%) and 2 (2.3%) of the 86 patients achieved CR, PR, and SD, respectively, as their best overall response. The ORR was 97.7% (95% CI 91.9–99.7). Supplementary Fig. 1a displays the CT images of a typical patient with the best overall response of CR.

**Fig. 3:**
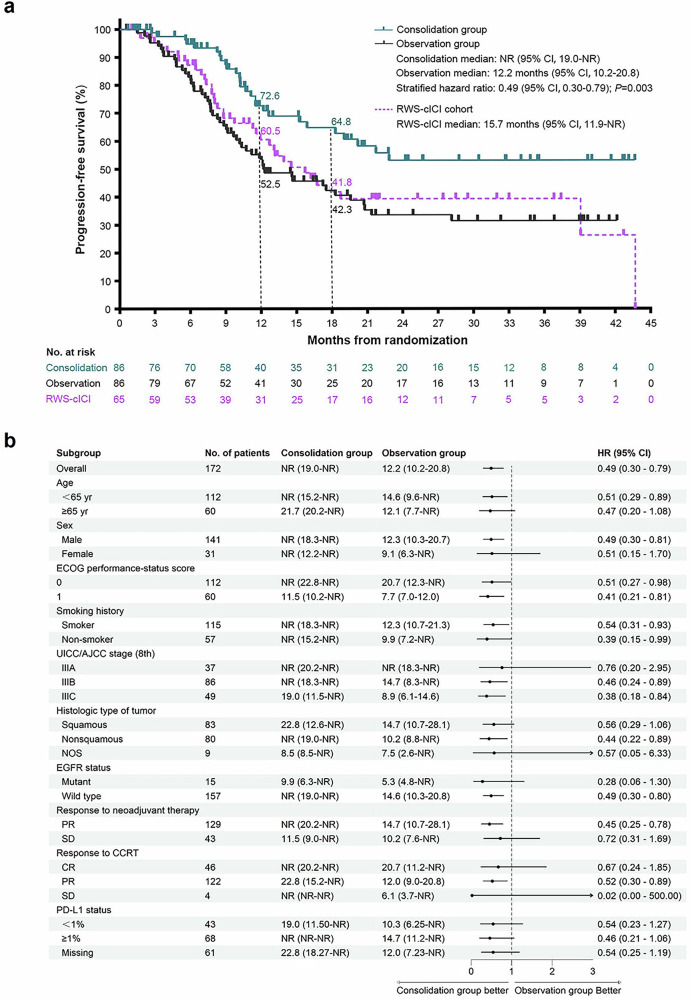
Progression-free survival in the randomized populations of CA209-7AL (*n* = 172) and RWS-cICI patients (*n* = 65). **a** Shows Kaplan‒Meier curves for progression-free survival (PFS) in the randomized CA209-7AL and RWS-cICI cohorts. The P value for CA209-7AL was calculated via a stratified two-sided log-rank test, with the hazard ratio and confidence intervals estimated according to a stratified Cox proportional hazards model with the treatment group as a covariate. **b** Shows the subgroup analyses for CA209-7AL. The unstratified data are reported. All subgroup analyses were prespecified. CCRT concurrent chemoradiotherapy, CR complete remission, ECOG PS Eastern Cooperative Oncology Group performance status, EGFR epidermal growth factor receptor, NOS not otherwise specified, PR partial remission, RWS-cICI cohort patients who received consolidative immunotherapy in a real-world study, SD stable disease

In the consolidation group, 27 of 86 patients experienced disease progression or died, whereas in the observation group, 51 of 86 patients experienced disease progression or died. PFS was significantly longer with consolidative NIVO than with observation (median not reached [NR] vs. 12.2 months [95% CI 10.2–20.8]; hazard ratio 0.49 [95% CI 0.30–0.79], *p* = 0.003; Fig. 3a). The 12-month and 18-month PFS rates were 72.6% (95% CI 62.5–84.4%) and 64.8% (95% CI 53.7–78.1%), respectively, in the consolidation group, whereas they were 52.5% (95% CI 42.6–64.8%) and 42.3% (95% CI 32.4–55.1%), respectively, in the observation group. A PFS benefit with consolidative NIVO compared with observation was detected across most prespecified subgroups (Fig. 3b).

The magnitude of PFS benefit with consolidative NIVO was greater for patients with stage IIIB (18-month PFS: 66.3% in the consolidative NIVO group vs. 44.9% in the observation group, *p* = 0.018) and IIIC disease (18-month PFS: 55·8% in the consolidative NIVO group vs. 16·5% in the observation group, *p* = 0.013) than for those with stage IIIA disease (18-month PFS: 78·7% in the consolidative NIVO group vs. 70·6% in the observation group, *p* = 0.694) (supplementary Fig. 2). The PFS benefit with consolidative NIVO was also greater in patients who achieved PR to neoadjuvant chemo-NIVO therapy (18-month PFS: 70.9% in the consolidative NIVO group vs. 43.7% in the observation group, *p* = 0.004) than in those with SD after neoadjuvant chemo-NIVO therapy (18-month PFS: 41.0% in the consolidative NIVO group vs. 37.7% in the observation group, *p* = 0.448) (supplementary Fig. 3).

The OS data were immature. There were 18 of 86 patients in the consolidation group and 34 of 86 patients in the observation group who died at the data cutoff. There was a trend toward improved OS with consolidative NIVO (median NR vs. NR, 18-month OS 73.4% vs. 61.0%, hazard ratio 0.56 [0.31–1.01], *p* = 0.050; supplementary Fig. 4).

In summary, consolidative NIVO demonstrated a significant and clinically meaningful improvement in PFS compared to observation, with the magnitude of benefit being particularly pronounced in patients with advanced disease stage (IIIB/C) and those achieving a PR to neoadjuvant therapy.

### Patterns of disease progression in a randomized population in CA209-7AL patients

At the data cutoff date, 17.4% (15/86) and 47.7% (41/86) of patients in the consolidation and observation groups, respectively, experienced disease progression. Locoregional only progression occurred in 8.1% (7/86) vs. 19.8% (17/86) of patients; distant only progression occurred in 4.7% (4/86) vs. 23.3% (20/86); and simultaneous locoregional/distant progression occurred in 4.7% (4/86) vs. 4.7% (4/86) of patients in the consolidation and observation groups, respectively. The median time to death or locoregional recurrence improved with consolidative NIVO versus observation (NR vs. NR; HR 0.48 [95% CI 0.23–0.99]; *p* = 0.044). The median time to death or distant progression was also improved with consolidative NIVO (NR vs. 21.3 months; HR 0.55 [95% CI, 0.32–0.96]; *p* = 0.032).

Supplementary Table 2 shows the detailed failure patterns. In patients with locoregional progression (*n* = 32), in-field progression accounted for 84.4% (27/32) of patients. In patients with distant progression (*n* = 32), oligometastasis accounted for 71.9% (23/32), and the most common distant site at progression was the brain (14/32). Among patients with disease progression, 80.0% (12/15) in the consolidation group and 80.5% (33/41) in the observation group received salvage treatment. The most commonly used salvage treatments were chemotherapy with or without immunotherapy (24/56, 42.9%) and radiotherapy (9/56, 16.1%) (supplementary Table 3). Supplementary Fig. 1b displays CT images of a patient who underwent salvage SBRT following local recurrence.

Collectively, consolidative NIVO significantly delayed both locoregional and distant recurrence, with the majority of progressed patients across both groups receiving subsequent salvage therapy.

### Safety in CA209-7AL patients

Adverse events (AEs) of any grade during the neoadjuvant period occurred in 98.1% (259/264) of patients (supplementary Table 4). The incidence of Grade (G) 3 or 4 adverse events was 45.5% (120/264). The most common G 3–4 adverse event was lymphopenia (93/264, 35.2%). G3 pneumonitis was observed in 1.1% (3/264) of the patients. Discontinuation of neoadjuvant chemo-NIVO therapy due to adverse events occurred in 7.2% of patients. No Grade 5 adverse events were recorded during the neoadjuvant period.

AEs of any grade during the CCRT period were observed in 98.3% (238/242) of the patients (supplementary Table 4). The incidence of G3 or G4 adverse events was 40.9% (99/242). The most common G 3–4 adverse event was lymphopenia (89/242, 36·8%). G3 esophagitis was observed in 3.3% (8/242) of the patients. G2+ pneumonitis was observed in 9.9% (24/242) of patients; among them, 2.5% (6/242) had G3 or G4 pneumonitis. No Grade 5 adverse events were recorded during the CCRT period. Discontinuation of CCRT due to adverse events occurred in 2.1% of patients.

AEs of any grade occurring after randomization were observed in 73.3% (63/86) of patients in the consolidation group and in 47.7% (41/86) of patients in the observation group (Table 2). G 3-4 AEs occurred in 9.3% of patients with consolidation and in 4.6% of patients with observation, including pneumonitis (2.3% with consolidation and 2.3% with observation) and proximal bronchial tree toxicity (3.5% with consolidation and 2.3% with observation). Treatment-related death occurred in 1 (1.2%) patient in the consolidation group because of pneumonitis. Discontinuation of consolidative NIVO due to adverse events occurred in 26.7% of patients in the consolidation group. Immune-mediated AEs of any grade were reported in 31.4% of patients in the consolidation group (supplementary Table 5). Two patients (2.3%) experienced grade 3 to 5 immune-mediated AEs, both of which were pneumonitis. Treatments for immune-mediated adverse events included systemic glucocorticoids (in 15.1% of patients), high-dose glucocorticoids (3.5%), endocrine therapy (5.8%) and other immunosuppressive therapies (1.2%). Two patients required hospitalization due to grade 3‒5 pneumonitis (2.3%).

**Table 2:** Adverse events after randomization in CA209-7AL (*N* = 172)

Adverse Event	No. (%)							
	Consolidation group (*N* = 86)				Observation group (*N* = 86)			
Led to discontinuation of consolidation	23 (26.7%)				----			
	Any Grade	Grade 3	Grade 4	Grade 5	Any Grade	Grade 3	Grade 4	Grade 5
All	63 (73.3%)	8 (9.3%)	0	1 (1.2%)	41 (47.7%)	4 (4.6%)	0	0
Pneumonitis	28 (32.6%)	2 (2.3%)	0	1 (1.2%)	14 (16.3%)	2 (2.3%)	0	0
Proximal bronchial tree toxicity	17 (19.8%)	3 (3.5%)	0	0	17 (19.8%)	2 (2.3%)	0	0
Cough	26 (30.2%)	2 (2.3%)	0	0	6 (7.0%)	2 (2.3%)	0	0
Dyspnea	11 (12.8%)	2 (2.3%)	0	0	5 (5.8%)	2 (2.3%)	0	0
Lymphopenia	20 (23.3%)	3 (3.5%)	0	0	9 (10.5%)	0	0	0
Hemoglobin decreased	9 (10.5%)	0	0	0	0	0	0	0
Alanine aminotransferase increased	10 (11.6%)	0	0	0	0	0	0	0
Aspartate aminotransferase increased	7 (8.1%)	0	0	0	0	0	0	0
Hyponatraemia	7 (8.1%)	0	0	0	0	0	0	0
Rash	6 (7.0%)	0	0	0	0	0	0	0
Hypothyroidism	5 (5.8%)	0	0	0	0	0	0	0

Included were events occurring in at least 5% patients in either group and all grade 3–5 treatment-related adverse events

Overall, consolidative NIVO following neoadjuvant chemo-NIVO and CCRT demonstrated a generally manageable toxicity profile.

### Treatment details of the RWS cohort

A total of 106 patients underwent CCRT, with 98 (98/106, 92.5%) completing CCRT and 65 (65/106, 61.3%) proceeding to cICI following CCRT. The most common reasons for not receiving cICI included treatment-related toxicity (14/106, 13.2%) and disease progression (12/106, 11.3%). Among the 65 patients who received cICI, 14 completed the treatment, 12 were still receiving cICI, and 39 discontinued cICI due to disease progression (*n* = 9), toxicity (*n* = 22), patients’ decisions (*n* = 4), or COVID-19-related reasons (*n* = 4). For those who either completed or discontinued cICI, the median number of cICI cycles was 5 (IQR, 2–12). Details regarding CCRT and cICI are provided in supplementary Table 6.

### Efficacy and safety results of the RWS cohort

For all the patients in the RWS, the ORR after CCRT was 75.5% (95% CI 66.2–83.3%). For patients who received consolidative immunotherapy following CCRT (the RWS-cICI cohort), the overall ORR was 90·8% (95% CI 81.0–96.5%) (Fig. 2).

For patients in the RWS-cICI cohort, the median duration of follow-up was 22.0 months (range, 1.0–43.7) (data cutoff on January 31, 2024). Thirty-five of 65 patients experienced disease progression or died. The median PFS was 15.7 months [95% CI 11.9-NA] (Fig. 3a). The 12-month and 18-month PFS rates were 60.5% (95% CI 49.1–74.7%) and 41.8% (95% CI 30.2–57.8%), respectively. Sixteen of the 65 patients died at the data cutoff, and the median OS was not reached (supplementary Fig. 4).

A total of 43.1% (28/65) of patients experienced disease progression. Locoregional only progression occurred in 29.2% (19/65) of the patients; distant only progression occurred in 10.7% (7/65); and simultaneous locoregional/distant progression occurred in 3.0% (2/65) of the patients. Supplementary Tables 2 and 3 show the detailed failure patterns and salvage treatments used.

PFS was greater in the NIVO consolidation group than in the RWS-cICI cohort (median NR vs. 15.7 months [95% CI, 11.9–NA]; hazard ratio: 0.57 [95% CI, 0.34–0.94]; *p* = 0.027). In contrast, the difference between the observation group and the RWS-cICI cohort was not statistically significant (median 12.2 months [95% CI, 10.2–20.8] vs. 15.7 months [95% CI, 11.9–NA]; hazard ratio: 1.16 [95% CI, 0.75–1.79]; *p* = 0.507). Additionally, we compared real-world PFS (rwPFS), measured from the time of screening, between all patients enrolled in CA209-7AL (*n* = 264) and those in the RWS (*n* = 106). The median rwPFS was 16.7 months (95% CI, 15.5–18.1) in the CA209-7AL study, whereas it was 14.7 months (95% CI, 12.2–18.2) in the RWS cohort (hazard ratio: 0.75 [95% CI, 0.56–0.99]; *P* = 0.043) (supplementary Fig 5).

In the RWS cohort, AEs of any grade during the CCRT period were observed in 98.1% (104/106) of the patients (supplementary Table 7). The incidence of G3 or G4 adverse events was 53.8% (57/106). The most common G3-4 adverse event was lymphopenia (55/106, 52.9%). G3 esophagitis was observed in 11.3% (12/106) of the patients. G2+ pneumonitis was observed in 17.0% (18/106) of patients, among whom 3.8% (4/106) had G3 or G4 pneumonitis. During cICI, AEs of any grade were observed in 76.9% (50/65) of patients. G3 AEs occurred in 15.4% (10/65) of patients, including G3 pneumonitis in 3.1% (2/65) of patients. No G4 or G5 adverse events were recorded.

Comparison between CA209-7AL and the RWS suggests that the integrated strategy of neoadjuvant chemo-NIVO followed by CCRT and consolidative NIVO confers a survival benefit over CCRT followed by consolidative immunotherapy, without increasing toxicity.

### NGS results

We obtained NGS results with qualified samples from 106 patients in CA209-7AL and 13 patients in the RWS cohort (supplementary Fig 6). The median TMB was 7.68 Muts/Mb (range, 0–121.0). The most commonly altered genes were *TP53* (91 [76.5%] patients), *LRP1B* (29 [24.4%]), *EGFR* (22 [18.5%]), *CDKN2A* (22 [18.5%]), *PIK3CA* (19 [16.0%]), and *FAT1* (17 [14.3%]). Hallmark pathway enrichment analysis revealed that the E2F target (69 [58.0%]), p53 (62 [52.1%]), and Wnt-β catenin signaling (58 [48.7%]) pathways were the most frequently altered cancer-related pathways.

We further evaluated TMB, individual somatic mutations, and pathways that may be associated with treatment outcomes in CA209-7AL patients (Supplementary Fig. 7). Patients with a high TMB tended to have a greater ORR to neoadjuvant chemo-NIVO (69.8% vs. 52.4%, Fisher’s exact test, *p* = 0.107). Conversely, patients with *EGFR*-activating mutations and *KEAP1* mutations tended to have a lower ORR to neoadjuvant chemo-NIVO (*EGFR*: 42.9% vs. 64.1%, Fisher’s exact test, *p* = 0.149; *KEAP1*: 36.4% vs. 62.1%, Fisher’s exact test, *p* = 0.116). In the consolidation group, patients with a high TMB had significantly longer PFS than those with a low TMB (NR vs. 15.2 months, *p* = 0.042). A shorter PFS was observed in patients with *EGFR*-activating mutations (8.5 months vs. NR, *p* = 0.079), alterations in the Wnt-β-catenin signaling pathway (12.6 months vs. NR, *p* = 0.098), or alterations in the p53 pathway (19.0 months vs. NR, *p* = 0.172), with marginal statistical significance.

### Post hoc analysis

To clarify whether the observed survival advantage in the NIVO consolidation group compared with the RWS-cICI cohort was attributable to the neoadjuvant chemo-NIVO component or the hypofractionated CCRT regimen, we performed an exploratory post hoc propensity score–matched analysis. A total of 65 patients with unresectable stage III NSCLC from our institutional database who received hypofractionated CCRT followed by cICI without prior neoadjuvant immunotherapy. These patients composed the hypoCCRT-cICI cohort. Baseline characteristics were well balanced between the hypoCCRT-cICI cohort, the NIVO consolidation group, and the RWS-cICI cohort (supplementary Table 9). The treatment details are summarized in supplementary Table 10.

As of the data cutoff date (January 31, 2024), the median follow-up duration was 24.1 months (range, 2.0–42.7) for the hypoCCRT-cICI cohort. The ORR was 96.9% (95% CI, 89.3–99.6). The median PFS in the hypoCCRT-cICI cohort was 20.2 months (95% CI, 17.0–not reached), whereas it was 15.7 months in the RWS-cICI cohort (HR, 0.74; 95% CI, 0.47–1.18; *p* = 0.212) and not reached in the NIVO consolidation group (HR, 1.36; 95% CI, 0.83–2.24; *p* = 0.225) (supplementary Fig 8). G3 AEs occurred in 13.8% (9/65) of patients during cICI, including G3 pneumonitis in 3·1% (2/65) and G3 proximal bronchial tree toxicity in 4·6% (3/65). No G4 or G5 AEs were observed (supplementary Table 11).

## Discussion

The CA209-7AL study represents the first randomized investigation to assess the feasibility and efficacy of neoadjuvant and consolidative NIVO in combination with CCRT in patients with unresectable stage III NSCLC. Interim analysis revealed that, for patients who had not progressed following neoadjuvant chemo-NIVO and CCRT, consolidative NIVO significantly improved PFS compared with observation. The median PFS from randomization was not reached in the consolidative NIVO group, whereas it was 12.2 months in the observation group (HR = 0.49, *p* = 0.003). This survival advantage was durable, with 64.8% of patients in the consolidative NIVO group remaining progression-free at 18 months compared with 42.3% in the observation group. The regimen demonstrated a generally manageable toxicity profile. Postrandomization, the incidence of G3 or higher treatment-related pneumonitis was comparable between the consolidative NIVO group and the observation group (3.5% vs. 2.3%). Moreover, in the RWS-cICI cohort, in which patients received CCRT followed by consolidative immunotherapy in routine clinical practice, the median PFS was 15.7 months, which was inferior to that of the consolidative NIVO group in CA209-7AL.

The PACIFIC trial demonstrated significantly prolonged PFS and OS with consolidative durvalumab compared with placebo (median PFS 16.8 vs. 5.6 months, median OS 47.5 vs. 29.1 months) following CCRT in unresectable stage III NSCLC patients.^8^ A phase III GEMSTONE-301 trial also revealed significant improvement in median PFS (9.0 months vs. 5.8 months) with sugemalimab over placebo.^16^ The PACIFIC-R study demonstrated the efficacy of consolidative durvalumab in real-world clinical practice, with a median PFS of 21.7 months.^17^ Other trials, such as COAST^18^ and BTCRC-LUN16-081,^19^ have shown promising PFS results with consolidative dual checkpoint inhibition. These studies provide a strong foundation for consolidative immunotherapy. However, they enrolled participants who had completed chemoradiation with preserved performance status and without progressive disease or major toxicities. In real-world clinical settings, a substantial proportion of patients with unresectable stage III NSCLC receiving CCRT as definitive treatment present with stage IIIB-C disease and compromised lung function. These patients face an elevated risk of disease progression, necessitate larger irradiation volumes during radiotherapy, and are more susceptible to radiation-induced pulmonary toxicity.^20,21^ This challenge underpins the rationale for our study of CA209-7AL, which aims to optimize the treatment strategy from the neoadjuvant phase onward and potentially enable more patients to benefit from immunotherapy. Given the encouraging results of neoadjuvant chemoimmunotherapy from the NADIM^2^ and CheckMate 816^4^ trials in patients with resectable NSCLC, neoadjuvant chemoimmunotherapy is expected to reduce the tumor burden and enhance immune priming prior to CCRT. However, it remains unclear whether consolidative immunotherapy could further enhance outcomes after effective neoadjuvant treatment and CCRT. In light of these considerations, we proposed this phase II clinical trial to evaluate the efficacy of neoadjuvant chemoimmunotherapy followed by CCRT and consolidative immunotherapy in patients with unresectable stage III NSCLC. The consolidation group was compared with an observation group and a real-world study (RWS) cohort utilizing the PACIFIC regimen.

Several studies have shown the promising efficacy of neoadjuvant immunotherapy followed by CCRT and consolidative immunotherapy in unresectable stage III NSCLC patients, with a 1-year PFS of 67.7–71.6%.^22–25^ However, these studies employed a single-arm design and lacked comparative data to determine whether consolidative immunotherapy provides additional benefits after effective neoadjuvant treatment and CCRT. Our randomized study indicated that consolidative NIVO following neoadjuvant chemo-NIVO and CCRT further enhanced treatment efficacy, improved local control and reduced distant recurrence. Despite the high ORR achieved in the two groups (98.8% in the consolidative NIVO group vs. 97.7% in the observation group), the 18-month PFS rate with observation was only 42.3%, whereas it was 64.8% in the consolidative NIVO group. Both the median time to death or locoregional recurrence and the median time to death or distant progression improved with consolidative NIVO versus observation. These results imply that consolidative immunotherapy remains crucial following neoadjuvant chemo-NIVO and CCRT in unresectable stage III NSCLC patients.

Subgroup analysis of CA209-7AL revealed that the median PFS was longer with consolidative NIVO than with observation following neoadjuvant chemo-NIVO and CCRT, irrespective of age, sex, smoking history, ECOG performance status, histology, disease stage, and PD-L1 status. In the CheckMate 77 T study, a more pronounced benefit of perioperative NIVO was observed in stage III NSCLC patients than in those with stage II disease.^5^ Consistent with these findings, our study demonstrated a greater PFS benefit with consolidative NIVO for patients with stage IIIB and IIIC disease than for those with IIIA disease. These findings suggest that patients with more advanced disease, who are at a greater risk of recurrence after neoadjuvant chemo-NIVO therapy and CCRT, derive significant benefit from consolidative immunotherapy.

CA209-7AL revealed that patients with partial remission to neoadjuvant chemo-NIVO exhibited superior 18-month PFS (70.9% in the consolidative NIVO group vs. 43.7% in the observation group) than did those with stable disease (41.0% in the consolidative NIVO group vs. 37.7% in the observation group). This might be attributed to the high proportion of stage IIIB-IIIC patients in our cohort, for whom CCRT served as the definitive treatment, making tumor volume reduction before CCRT critical for combination regimen success. Furthermore, we observed a greater benefit of consolidative NIVO in patients who achieved partial remission to neoadjuvant chemo-NIVO than in those with stable disease after neoadjuvant chemo-NIVO, suggesting that the initial response to neoadjuvant chemo-NIVO therapy might serve as an early indicator of consolidative NIVO efficacy.

Although the CA209-7AL study did not include a direct control group treated with the PACIFIC regimen, the comparison between the two CA209-7AL groups and the RWS cohort was informative. In the CA209-7AL study, neoadjuvant chemo-NIVO achieved substantial tumor volume reduction, with an ORR of 65.5%. Combining neoadjuvant chemo-NIVO with CCRT achieved an ORR of 84.3% (95% CI 79.1–88.6%) from baseline, which was superior to the ORR of 75.5% (95% CI, 66.2–83.3%) observed in the RWS cohort. Notably, PFS in the consolidation group was superior to that in the RWS-cICI cohort (median PFS not reached vs. 15.7 months), potentially due to substantial tumor burden reduction and enhanced immune priming prior to CCRT. However, the RWS-cICI cohort demonstrated better survival outcomes than did the observation group did (median PFS: 15.7 vs. 12.2 months). This may be attributed to the greater risk of distant metastases in the observation group, which did not receive consolidative immunotherapy (27.9% [24/86] in the observation group vs. 13.8% [9/65] in the RWS-cICI cohort). These findings suggest that a strong initial response to neoadjuvant chemoimmunotherapy does not necessarily translate into improved long-term survival. Consolidative immunotherapy plays a pivotal role in enhancing outcomes and reducing distant recurrences in unresectable stage III NSCLC, even after effective neoadjuvant treatment.

In the CA209-7AL study, we employed a hypofractionated radiotherapy regimen followed by a hypofractionated boost to enhance local control and optimize synergy with immunotherapy. Previous studies have suggested that hypofractionated chemoradiotherapy (hypo-CCRT) may improve local control while maintaining a favorable safety profile in patients with stage III NSCLC.^26,27^ When combined with immunotherapy, hypo-CCRT offers advantages over conventional fractionation, including enhanced immunogenic effects and better preservation of circulating lymphocytes due to fewer dose fractions.^28–31^ Our previous study demonstrated that a regimen of 60 Gy delivered in 12–20 fractions, consisting of an initial course followed by a boost course, could be safely combined with consolidative immunotherapy.^32^ Post hoc analysis comparing the NIVO consolidation group, RWS-cICI cohort, and hypoCCRT-cICI cohort revealed that hypo-CCRT was associated with a trend toward improved PFS compared with conventional fractionation when used with consolidative immunotherapy. Furthermore, the incorporation of neoadjuvant chemoimmunotherapy prior to hypo-CCRT conferred additional PFS benefits beyond those achieved with hypo-CCRT alone. The three groups presented comparable toxicity rates. These findings support the efficacy and feasibility of hypo-RT combined with concurrent chemotherapy and consolidative immunotherapy. However, prospective data on hypo-CCRT with consolidative immunotherapy remain limited.^33^ Notably, we observed a greater incidence of proximal bronchial tree toxicity in the hypoCCRT-cICI cohort than in the RWS-cICI cohort. Therefore, before this approach is routinely implemented for locally advanced NSCLC, careful consideration and additional clinical research, including randomized controlled trials, are essential to establish robust supporting evidence.

The incidence of in-field local recurrence in CA209-7AL patients was lower in both the consolidation (8.1%) and observation (20.9%) groups than in the RWS cohort (29.9%). This enhanced local control could be attributed to the tumor shrinkage achieved through neoadjuvant chemo-NIVO therapy and hypo-CCRT. Notably, we found a predominance of oligometastasis in patients who developed distant metastasis across both the consolidation (6/8) and observation groups (17/24), with the brain being the most common site of distant progression. For these patients, local ablative therapy and palliative chemotherapy/immunotherapy could still prolong survival. Consequently, the OS outcomes in CA209-7AL patients are as expected, although they remain immature at this stage.

Pneumonitis is a common adverse event associated with the administration of immunotherapy and CCRT. G2+ pneumonitis has been reported in 10–25% of patients, and G3+ pneumonitis has been reported in 0–7% of patients receiving single-agent consolidative immunotherapy after CCRT.^34^ Concurrent administration of immunotherapy and CCRT led to increased pulmonary toxicity, with the incidence of G3+ pneumonitis reported to be 8.0% and 6.9% in cohorts A and B of the KEYNOTE-799 trial^23^ and 12% in the NICOLAS study.^35^ In the CA209-7AL study, the incidence of pneumonitis after CCRT remained low, with a G2+ rate of 9.9%, whereas it was 17.0% in the RWS cohort. This reduction can be attributed to substantial tumor shrinkage after neoadjuvant chemo-NIVO. After randomization, the incidence of pneumonitis of any grade was greater with consolidative NIVO than with observation (32.6% vs. 16.3%). Most cases were of low grade and manageable. Overall, the incidence of G3+ pneumonitis was 3.5% with consolidative NIVO, which is consistent with the established toxicity profiles of immunotherapy following CCRT for stage III NSCLC.

CCRT is known to induce severe lymphopenia, which can persist for weeks and is associated with an increased risk of immune-related adverse events and reduced efficacy of immunotherapy.^36,37^ Emerging data have highlighted the prognostic value of circulating lymphocyte counts prior to initiating consolidative immunotherapy after CCRT in patients with locally advanced NSCLC.^38,39^ Previous studies have shown that most patients recover from severe lymphopenia approximately two months after CCRT.^40^ Therefore, the current study design incorporated a two-month interval between the completion of CCRT and the initiation of immunotherapy to allow for lymphocyte recovery. While the PACIFIC trial demonstrated benefits with earlier initiation of consolidative immunotherapy, our trial prioritized minimizing adverse events, particularly given the added complexity of combining neoadjuvant chemoimmunotherapy with CCRT.

To identify potential predictive biomarkers, we analyzed the TMB and individual somatic mutations associated with treatment outcomes in CA209-7AL patients. Substantial evidence supports the association between TMB and responsiveness to immunotherapy.^41,42^ Similarly, our analysis revealed that patients with a high TMB demonstrated prolonged PFS with consolidative NIVO following neoadjuvant chemo-NIVO and CCRT. Furthermore, we observed that patients with EGFR-activating mutations tended to have shorter PFS with consolidative NIVO, which is consistent with prior studies.^43,44^ Notably, the associations between individual mutations or pathways and treatment outcomes were mostly marginally significant, reinforcing the concept that effective subtyping may require the integration of multiple genomic markers rather than relying on a single feature.

A key limitation of the CA209-7AL study is the absence of an internal control arm receiving CCRT followed by consolidative immunotherapy. Instead, this treatment approach was evaluated in a parallel RWS cohort. The radiotherapy protocols differed in the RWS cohort from those used in the CA209-7AL trial, introducing potential confounding variables. While the study was adequately powered to assess the efficacy of consolidative nivolumab within the CA209-7AL population, the statistical design did not support robust cross-cohort comparisons between the trial arms and the RWS cohort. Another limitation of CA209-7AL is the small sample size in certain subgroups, such as females (*n* = 31), patients with tumors of NOS histology (*n* = 9), and those with stable disease after CCRT (*n* = 4). The lack of observed benefit in these subgroups may be influenced by the limited number of participants, which could reduce the statistical power to detect meaningful differences and potentially underestimate the treatment effect. For example, unlike our findings, the PACIFIC study demonstrated a significant PFS benefit in females and patients with stable disease after CCRT. Further research with larger patient cohorts is needed to validate these results. Additionally, the assessment of PD-L1 expression was not mandatory for study enrollment, and PD-L1 expression could not be tested in nearly half of the enrolled patients because of limited biopsy tissue. Furthermore, 8.3% of the enrolled patients had EGFR-mutated tumors, as the optimal treatment strategy for patients with EGFR-mutated tumors was not well established at the time of trial initiation. However, recent findings from the Laura study demonstrated the efficacy of consolidative osimertinib following CCRT.^45^

In conclusion, the interim analysis of this randomized, phase II trial, CA209-7AL, for patients with unresectable stage III NSCLC demonstrated a significant improvement in PFS with consolidative NIVO compared with observation following neoadjuvant chemotherapy plus NIVO and CCRT. This improvement was also observed compared with the parallel RWS-cICI cohort, in which patients received CCRT followed by consolidative immunotherapy in routine clinical practice. Patients with a high TMB experienced a greater ORR after neoadjuvant chemo-NIVO and demonstrated prolonged PFS with consolidative NIVO following neoadjuvant chemo-NIVO and CCRT. Extended follow-up is essential to validate these findings, and future studies directly comparing this regimen with the PACIFIC diagram are needed.

## Materials and methods

### Study design and patients

CA209-7AL is a randomized, multicenter, phase II study that was conducted at 5 hospitals or academic research centers in China. The eligibility criteria for initial neoadjuvant chemo-NIVO therapy included age 18 to 75 years; previously untreated NSCLC with unresectable stage III (IIIA, IIIB, or IIIC) disease according to the American Joint Committee on Cancer version 8 (AJCC 8th) staging system; Eastern Cooperative Oncology Group performance status of 0 or 1; measurable disease per Response Evaluation Criteria in Solid Tumors, version 1.1 (RECIST v1.1); and adequate organ function. Patients were excluded if they had any of the following: prior exposure to any anti-PD-1 or anti-PD-L1 antibody, a history of primary immunodeficiency, an active autoimmune disease requiring systemic treatment, or an active infection requiring systemic therapy. Eligibility for randomization required the completion of initial neoadjuvant chemo-NIVO therapy and CCRT within 2 months, no disease progression, an ECOG PS of 0 to 1 at randomization, adequate organ function, and the absence of any unresolved G2+ toxicity from prior therapy before random assignment. The full eligibility criteria are described in the protocol in Supplement 1. The study is registered with ClinicalTrials.gov (identifier: NCT04085250; registered September 9, 2019). Supplementary Fig. 9 shows a schematic diagram of the study design.

Patients who met the eligibility criteria but declined to participate in CA209-7AL were included in the parallel RWS. These patients received standard-of-care CCRT followed by consolidative immunotherapy.

### Procedures of CA209-7AL

#### Neoadjuvant chemotherapy and nivolumab

Eligible patients received neoadjuvant chemo-NIVO therapy comprising docetaxel 60 mg/m^2^ d1, cisplatin 25 mg/m^2^ d1–3 and NIVO 360 mg d1, which was administered intravenously every 3 weeks for a total of 2 cycles.

#### Concurrent chemoradiotherapy

(CCRT) was initiated 21 to 28 days after the second cycle of neoadjuvant chemo-NIVO therapy in patients without distant progression or persistent toxicity. The total dose of radiotherapy was 54–64 Gy in 12–16 daily fractions, delivered via intensity-modulated radiation therapy (IMRT) and esophagus-sparing techniques,^46^ with daily cone-beam CT for position verification. Radiation plans were centrally reviewed for quality assurance. Additional thoracic radiotherapy details, including dose constraints for normal tissues, are provided in supplementary Table 8, and the study protocol is provided in Supplement 1. Concurrent chemotherapy consisted of weekly intravenous docetaxel (25 mg/m^2^, d1) and cisplatin (25 mg/m^2^, d1).

#### Randomization and masking

Patients with no disease progression or any unresolved G2+ toxicity following neoadjuvant chemo-NIVO therapy and CCRT were randomly assigned (1:1) via a block size of six to receive NIVO 360 mg intravenously every 3 weeks or for observation. Randomization was performed at 2 months after the completion of CCRT and was stratified by age, sex, smoking history and EGFR mutation status. The study coordinator managed the centralized randomization process, with investigators contacting the coordinator to receive patient allocations. The allocation sequence remained confidential and was only known by the study coordinator until the interventions were assigned. The investigators and patients were aware of the treatment group assignments, whereas two radiologists conducted blinded reviews of the imaging data.

#### Consolidative Nivolumab

Administration of consolidative NIVO commenced within 3 days following randomization to NIVO and continued for a maximum duration of 12 months. It was discontinued prior to 12 months if there was confirmed disease progression, unacceptable toxicity, withdrawal of consent, intercurrent illness or the investigator’s decision.

#### Assessments

Tumors were assessed at baseline, 2 weeks after neoadjuvant chemo-NIVO therapy, 2 months after CCRT and then every 3 months during consolidation or observation until disease progression or death, whichever came first. Tumor response was defined as CR, PR, SD or PD according to RECIST version 1.1 (recorded as the aggregate response to former therapy). AEs were evaluated at baseline, during the treatment period, and at the safety follow-up visit and were graded with CTCAE, version 5.0.

### Procedures of the RWS

Patients in the RWS underwent standard-of-care CCRT, which consisted of a total radiation dose of 60–64 Gy delivered in 30–32 fractions concurrently with platinum-based chemotherapy. This was followed by consolidative PD-1/PD-L1 inhibitors, which were initiated within two months after the completion of CCRT. Regular follow-up and data collection were performed.

### Endpoints

For CA209-7AL patients, the primary endpoint was PFS (according to RECIST 1.1). PFS was defined as the time from randomization to the date of the first documented event of tumor progression or death from any cause. The secondary endpoints included OS, ORR and AEs. OS was defined as the time from randomization until death from any cause. The ORR was defined as the sum of the rates of CR and PR. AEs during the neoadjuvant period included those occurring from the first neoadjuvant dose to the initiation of CCRT. AEs during the CCRT period included those occurring from the initiation of CCRT to the time of randomization. Postrandomization AEs in the randomized population included those occurring after the time of randomization.

In the RWS, the primary endpoint was PFS. The secondary endpoints included OS, ORR and AEs. PFS and OS were calculated from the time of the first dose of consolidative immunotherapy. AEs during the CCRT period included those occurring from the initiation of CCRT until the start of consolidative immunotherapy. AEs during the consolidative period included those occurring after the initiation of consolidative immunotherapy.

### Biomarker analysis

Pretreatment biopsy samples from enrolled patients were collected to evaluate the potential impact of PD-L1 expression and somatic mutations on treatment outcomes. PD-L1 expression was assessed via the PD-L1 immunohistochemical 22C3 pharmDx assay and quantified via the tumor proportion score (TPS). Next-generation sequencing (NGS) was performed via the Gene+ Seq-2000 sequencing system with a panel encompassing 1021 cancer-associated genes.^47^ Simultaneously, matched peripheral white blood cell DNA was sequenced to filter out benign single nucleotide polymorphisms and potential germline mutations. TMB was classified as high (TMB-H) for samples with >10 mutations/Mb. Genes with identified somatic variants were further analyzed and grouped into functional pathways via the Molecular Signatures Database (MSigDB) hallmark gene sets.^48^

### Statistical analysis

CA209-7AL was estimated to have a power of 90% to detect an improvement in the median PFS from 10 months^49^ to 18 months, corresponding to a hazard ratio of 0.56, using a log-rank test with a two-sided type 1 error of 0.05. With 2 years of accrual and 2 years of follow-up, the number of patients needed for randomization was 150 (75 per group), and the number of PFS events needed for the final analysis was 123. With a 10% dropout rate considered, a total of 168 patients (84 per group) were needed for randomization. We assume that (1) approximately 15% of patients who have received neoadjuvant chemo-NIVO therapy will not receive subsequent CCRT and that (2) approximately 25% of patients who have received CCRT will not be eligible for randomization. Therefore, 264 patients in total need to be enrolled in neoadjuvant chemo-NIVO therapy. We assumed one planned interim analysis when approximately 60% of events had occurred. The threshold of significance, defined by the O’Brien-Fleming type boundary, was 0.0076 in the interim analysis and 0.048 in the final analysis.

In CA209-7AL patients, PFS and OS were assessed in all randomized patients. The ORR to neoadjuvant chemo-NIVO therapy was assessed in patients who had received at least 1 cycle of neoadjuvant chemo-NIVO therapy. The ORR to CCRT was assessed in patients who underwent CCRT. The overall ORR with consolidative NIVO or observation was assessed in patients who were randomized. Patients with missing ORR data were considered nonresponders. AEs during the neoadjuvant chemo-NIVO therapy period were evaluated in patients who received at least 1 cycle of neoadjuvant chemo-NIVO therapy. AEs during the CCRT period were assessed in patients who received at least 1 fraction of RT. AEs after randomization were assessed in all patients who received at least 1 cycle of consolidative NIVO or who received observation. PFS and OS were evaluated via the Kaplan‒Meier method. Between-group comparisons were performed via a log-rank test, stratified according to age, sex, smoking history and EGFR mutation status. HR and 95% CIs were calculated via a stratified Cox regression model. Prespecified key subgroup analyses (age [<65 years or ≥65 years], sex [male or female], ECOG performance status [0 or 1], smoking history [smoker or nonsmoker], disease stage [IIIA, IIIB or IIIC]), histology type [squamous, nonsquamous or NOS], EGFR status [mutant or wild type], PD-L1 status [<1%, ≥1% or missing], response to neoadjuvant chemo-NIVO therapy [PR or SD], response to CCRT [CR, PR or SD]) for PFS were performed, in which HRs and 95% CIs were calculated via an unstratified Cox regression model. Response rates were estimated via the Clopper‒Pearson method. In the RWS, PFS and OS were assessed in patients who received at least one infusion of consolidative immunotherapy. The ORR to CCRT was assessed in patients who underwent CCRT. The overall ORR was assessed in patients who received at least one infusion of consolidative immunotherapy. AEs during the CCRT period were assessed in patients who received at least 1 fraction of RT. AEs during the consolidative period were assessed in all patients who received at least 1 cycle of consolidative immunotherapy. As a post hoc analysis, we identified patients with unresectable stage III NSCLC who received hypofractionated CCRT followed by cICI without prior neoadjuvant immunotherapy from our institutional database between December 2019 and August 2023 (hypoCCRT-cICI cohort). We conducted 1:1 propensity score matching with the RWS-cICI cohort via a logistic regression model that included age, sex, smoking history and EGFR mutation status. Matching was performed with a caliper width of 0.25. Statistical analyses were performed via SPSS software (version 25.0) and R (version 3.6.1).

## Supplementary information

Supplement 1. Study Protocol of CA209-7AL

Supplement 2. Supplementary Tables and figures

## Data Availability

The processed sequencing data reported in this paper have been deposited in OMIX, China National Center for Bioinformation/Beijing Institute of Genomics, Chinese Academy of Sciences (https://ngdc.cncb.ac.cn/omix: accession no. OMIX011275). The original data supporting the results of this paper had been deposited in the Research Data Deposit repository (https://www.researchdata.org.cn: accession no. RDDA2025349690).
