# Supplementary material for: Consolidative nivolumab versus observation in unresectable stage III non-small cell lung cancer patients following neoadjuvant nivolumab plus chemotherapy and concurrent chemoradiotherapy (CA209-7AL): a randomized clinical trial
Source: Signal Transduct Target Ther. 2025 Sep 29;10:317. doi: 10.1038/s41392-025-02408-3 (PMC12477293; doi:10.1038/s41392-025-02408-3)
Supplement: Supplementary file 1 — Supplement 1. Study Protocol of CA209-7AL [file 41392_2025_2408_MOESM1_ESM.docx]

**A Phase II, Randomized Study of Nivolumab as Consolidation Therapy in Patients with Locally Advanced, Unresectable Non-Small Cell Lung Cancer (Stage III) Who Have Not Progressed Following** **Neoadjuvant Chemotherapy plus Nivolumab and Definitive Concurrent Chemoradiation Therapy**

# Study Protocol

# ISR CA209-7AL

# PI: Li Zhang, Hui Liu

Li Zhang, M.D., Ph.D., Department of Medical Oncology, 651 Dongfengdong Road, Sun Yat-sen University Cancer Center, Guangzhou, Guangdong, P.R. China. Zip code: 510060

Tel: +86 20 87343458 E-mail address: [zhangli@sysucc.org.cn](mailto:zhangli@sysucc.org.cn)

Hui Liu, M.D., Ph.D., Department of Radiation Oncology, 651 Dongfengdong Road, Sun Yat-sen University Cancer Center, Guangzhou, Guangdong, P.R. China. Zip code: 510060

Tel: +86 20 87343031 E-mail address: [liuhui@sysucc.org.cn](mailto:liuhui@sysucc.org.cn)

**Study synopsis**

**Background**

The prognosis of locally advanced non-small cell lung cancer remains poor, with a median survival time of 15 to 18 months. Radiotherapy combined with immunotherapy as a new combined treatment strategy is expected to further improve the survival of locally advanced NSCLC. Neoadjuvant immunotherapy has a certain theoretical basis, and has shown a preliminary good effect in operable NSCLC. From the results of PACIFIC and lun14-179 studies, immunotherapy is likely to be an excellent choice for consolidation treatment of locally advanced NSCLC patients after chemoradiotherapy. Therefore, we plan to conduct a phase II, randomized trial to explore the efficacy and safety of Nivolumab as consolidation therapy in patients with locally advanced, unresectable NSCLC who have not progressed following neoadjuvant chemotherapy plus Nivolumab and definitive concurrent chemoradiation.

**Study objectives**

- Primary Objective
- To assess the efficacy of Nivolumab consolidation compared with observation in terms of PFS.
- Secondary Objective
- To assess the efficacy of Nivolumab consolidation compared with observation in terms of OS and ORR
- To assess the safety of Nivolumab consolidation compared with observation in terms of AE
- To assess the symptoms and health-related quality of life in patients treated with Nivolumab consolidation compared with observation
- To assess the efficacy of neoadjuvant chemotherapy plus nivolumab in terms of ORR after neoadjuvant therapy
- To assess the efficacy of neoadjuvant chemotherapy plus nivolumab in terms of OS and PFS for all patients

**Study design**

This is a Phase II, randomized, multi-center study assessing the efficacy and safety of Nivolumab compared with observation, as sequential therapy in patients with locally advanced, unresectable NSCLC (Stage III), who have not progressed following neoadjuvant chemotherapy plus Nivolumab and definitive, platinum-based, concurrent chemoradiation therapy.

Approximately 264 patients will be enrolled and receive neoadjuvant chemotherapy plus Nivolumab. Approximately 224 patients will receive definitive, concurrent chemoradiation (patients will be suitable for definitive concurrent chemoradiation following neoadjuvant therapy). Approximately 168 patients will be randomized (patients will be in CR, PR or have SD following definitive, platinum-based, concurrent chemoradiation therapy) at 5 sites in China, in a 1:1 ratio (Nivolumab to observation) to 1 of 2 arms: • Nivolumab (360 mg every 3 weeks [Q3W] intravenous [iv] for up to 12 months) • observation. Randomization will be stratified by: age at randomization (<65 versus ≥65 years of age), sex (male versus female), smoking history (smoker versus non-smoker) and EGFR mutation status (wild type versus mutant).

**Duration of study**

The duration of this study is expected to be 4 years. We expect 2 years to enroll patients and 2 years to follow up patients.

**Eligibility**

**Inclusion criteria**

For inclusion in neoadjuvant therapy, patients should fulfil the following criteria:

- Provision of signed, written and dated informed consent prior to any study specific procedures；
- Male or female aged 18 years to 75 years；
- Patients must have histologically- or cytologically-documented NSCLC who present with locally advanced, unresectable (Stage III) disease;
- Without prior chemotherapy, radiotherapy, surgery, targeted therapy or immunotherapy;
- Tumor sample requirements: Mandatory provision of an unstained, archived tumor tissue sample in a quantity sufficient to allow for analysis;
- A recent tumor biopsy (taken following completion of the most recent therapy) is an optional requirement, provided that a biopsy procedure is technically feasible and the procedure is not associated with unacceptable clinical risk;
- Life expectancy ≥12 weeks;
- World Health Organization (WHO) Performance Status of 0 or 1;
- Evidence of post-menopausal status, or negative urinary or serum pregnancy test for female pre-menopausal patients within 14 days before the use of study drug (HCG has a minimum sensitivity of 25 IU/L or equivalent);
- Women must be non-breastfeeding
- Women of reproductive age (WOCBP) must agree to comply with the contraceptive method during the study nivolumab treatment and for a period of 5 months following the last administration of the study treatment (i.e., 30 days [ovulation cycle] plus approximately 5 half-lives of the study drug).
- Men who have sex with WOCBP must agree to comply with the contraceptive method during the study nivolumab treatment and for 7 months after the last administration of the study treatment (i.e. 90 days [sperm renewal cycle] plus approximately 5 half-life of the study drug).
- Spermless men do not have to comply with contraceptive requirements. WOCBP who continues to be asexual with the opposite sex does not have to comply with contraceptive requirements, but must still undergo the pregnancy tests described in this section.
- Adequate organ and marrow function as defined below:
  - Forced expiratory volume in 1 second (FEV1) ≥800ml
  - Absolute neutrophil count >1.5 x 10^9^/L (1500 per mm3)
  - Platelets >100 x 10^9^/L (100,000 per mm3)
  - Haemoglobin≥9.0 g/dL (5.59 mmol/L)
  - Serum creatinine CL >50 mL/min by the Cockcroft-Gault formula (Cockcroft and
- Gault 1976)
  - Serum bilirubin ≤1.5 x upper limit of normal (ULN).
  - AST and ALT ≤2.5 x ULN

**Exclusion criteria**

Exclusion criteria for enrolment for neoadjuvant therapy

Patients should not enter the study if any of the following exclusion criteria are fulfilled:

- Concurrent enrolment in another clinical study, unless it is an observational(non-interventional) clinical study;
- Mixed small cell and non-small cell lung cancer histology;
- Current or prior use of immunosuppressive medication within 28 days before the first dose of Nivolumab, with the exceptions of intranasal and inhaled corticosteroids or systemic corticosteroids at physiological doses, which are not to exceed 10 mg/day of prednisone, or an equivalent corticosteroid. Systemic steroid administration required to manage toxicities arising from radiation therapy delivered as part of the chemoradiation therapy for locally advanced NSCLC is allowed.
- Prior exposure to any anti-PD-1 or anti-PD-L1 antibody;
- Recent major surgery within 4 weeks prior to entry into the study (excluding the placement of vascular access) that would prevent administration of nivolumab;
- Active or prior documented autoimmune disease within the past 2 years;
- Active or prior documented inflammatory bowel disease (eg. Crohn’s disease, ulcerative colitis);
- History of primary immunodeficiency;
- History of organ transplant that requires therapeutic immunosuppression;
- Mean QT interval corrected for heart rate (QTc) ≥470 ms calculated from3 electrocardiograms (ECGs) using Bazett’s Correction;
- Uncontrolled intercurrent illness including, but not limited to, ongoing or active infection, symptomatic congestive heart failure, uncontrolled hypertension, unstable angina pectoris, cardiac arrhythmia, active peptic ulcer disease or gastritis, active bleeding diatheses including any patient known to have hepatitis B, hepatitis C or human immunodeficiency virus (HIV), or psychiatric illness/social situations that would limit compliance with study requirements or compromise the ability of the patient to give written informed consent;
- Known history of tuberculosis, except for obsolete pulmonary tuberculosis;
- Receipt of live attenuated vaccination within 30 days prior to study entry or within30 days of receiving nivolumab;
- History of another primary malignancy within 5 years prior to starting nivolumab, except for adequately treated basal or squamous cell carcinoma of the skin or cancer of the cervix in situ and the disease under study;
- Female patients who are pregnant, breast-feeding or male or female patients of reproductive potential who are not employing an effective method of birth control;
- Any condition that, in the opinion of the investigator, would interfere with evaluation of the nivolumab or interpretation of patient safety or study results.

### Exclusion criteria for concurrent chemoradiation following neoadjuvant therapy

Patients should not enter the concurrent chemoradiation phase if any of the following exclusion criteria are fulfilled:

- Patients who develop distant metastasis;
- Patients who develop locoregional disease progression and the irradiation dose of normal tissue will exceed the limit as defined in Section 7.
- World Health Organization (WHO) Performance Status of 2-4;
- Inadequate organ and marrow function as defined below:
  - Forced expiratory volume in 1 second (FEV1) <800ml
  - Absolute neutrophil count <1.5 x 10^9^/L (1500 per mm3)
  - Platelets <100 x 10^9^/L (100,000 per mm3)
  - Haemoglobin<9.0 g/dL (5.59 mmol/L)
  - Serum creatinine CL <50 mL/min by the Cockcroft-Gault formula (Cockcroft and
- Gault 1976)
  - Serum bilirubin >1.5 x upper limit of normal (ULN).
  - AST and ALT >2.5 x ULN

Further exclusion criteria for randomization into Nivolumab consolidation or observation group

Patients should not enter the randomization if any of the following exclusion criteria are fulfilled:

- Patients who have progressed whilst definitive platinum based, concurrent chemoradiation therapy;
- Current or prior use of immunosuppressive medication within 28 days before the first dose of Nivolumab, with the exceptions of intranasal and inhaled corticosteroids or systemic corticosteroids at physiological doses, which are not to exceed 10 mg/day of prednisone, or an equivalent corticosteroid. Systemic steroid administration required to manage toxicities arising from radiation therapy delivered as part of the chemoradiation therapy for locally advanced NSCLC is allowed.
- Any unresolved toxicity CTCAE >Grade 2 from the prior chemoradiation therapy will be excluded from randomization;
- Patients with Grade ≥2 pneumonitis from prior chemoradiation therapy will be excluded from randomization;
- Any prior Grade ≥3 immune-related adverse event (irAE) while receiving any previous immunotherapy agent, or any unresolved irAE>Grade 1.

**Treatment plan**

- Neoadjuvant therapy before radiotherapy

The neoadjuvant therapy before radiotherapy comprised of Docetaxel 60 mg/m^2^ d1 + Cisplatin 25 mg/m^2^ d1-3+Nivolumab 360 mg d1, Q3W, for a total of 2 cycles.

- Chemotherapy concurrent with radiotherapy

Docetaxel 25 mg/m^2^for 1 hour +Cisplatin 25 mg/m^2^, QW.

- Radiotherapy

A total dose of 60~64Gy in 12-16 daily fractions.

- Nivolumab consolidation or observation

Patients will receive either Nivolumab consolidation (360 mg) via iv infusion Q3W or observation after the chemo-radiotherapy. Administration of nivolumab will commence on Day 1 following randomization to Nivolumab after confirmation of eligibility and will continue on a Q3W schedule for a maximum duration of 12 months.

**Schedule of assessment**

The schedule for assessments at screening and during the Treatment Period is presented in Table 3. The schedule of study procedures during follow-up for patients who have completed study drug and achieved disease control (until confirmed PD) and patients who have discontinued study drug due to toxicity or a reason other than confirmed PD is presented in Table 4. The schedule of study procedures during follow-up for patients who have discontinued study drug due to confirmed PD is presented in Table 5.

**Statistics**

The primary endpoint is PFS. The primary statistical analysis of the efficacy of Nivolumab consolidation versus placebo will include all randomized patients, regardless of the treatment actually received.

**Table of contents**

[Study Protocol 1](#_Toc178150390)

[ISR CA209-7AL 1](#_Toc178150391)

[PI: Li Zhang, Hui Liu 1](#_Toc178150392)

[Exclusion criteria for concurrent chemoradiation following neoadjuvant therapy 4](#_Toc178150393)

[1. Background 8](#_Toc178150394)

[1.1 Overview of the treatment of locally advanced non-small cell lung cancer 8](#_Toc178150395)

[1.2 Immunotherapy combined with radiotherapy 8](#_Toc178150396)

[1.3 Advances in neoadjuvant immunotherapy for non-small cell lung cancer 8](#_Toc178150397)

[1.4 Optimizing the Dose of Radiotherapy 9](#_Toc178150398)

[2. Study Objectives 10](#_Toc178150399)

[3. Ethical Considerations 11](#_Toc178150400)

[4. Informed Consent 11](#_Toc178150401)

[5. Study Design 12](#_Toc178150402)

[5.1 Study design scheme 12](#_Toc178150403)

[5.2 Investigational products 13](#_Toc178150404)

[5.2.1 Identification of investigational products 13](#_Toc178150405)

[5.2.2 Storage of investigational products and use conditions 13](#_Toc178150406)

[5.3 Patient enrolment 13](#_Toc178150407)

[5.4 Multi-Disciplinary Discussion 13](#_Toc178150408)

[5.5 Procedures for Randomization 13](#_Toc178150409)

[5.6 Duration of study 14](#_Toc178150410)

[6. Eligibility 14](#_Toc178150411)

[6. 1 Inclusion criteria 14](#_Toc178150412)

[For inclusion in neoadjuvant therapy, patients should fulfil the following criteria: 14](#_Toc178150413)

[6.2 Exclusion criteria 15](#_Toc178150414)

[6.2.1 Exclusion criteria for enrolment for neoadjuvant therapy 15](#_Toc178150415)

[6.2.2 Exclusion criteria for concurrent chemoradiation following neoadjuvant therapy 15](#_Toc178150416)

[6.2.3 Further exclusion criteria for randomization into Nivolumab consolidation or observation group 16](#_Toc178150417)

[6.3 Withdrawal from study 16](#_Toc178150418)

[7. Treatment plan 16](#_Toc178150419)

[7.1 Neoadjuvant therapy before radiotherapy 16](#_Toc178150420)

[7.2 Chemotherapy concurrent with radiotherapy 17](#_Toc178150421)

[7.3 Adverse effect of chemotherapy and modification 17](#_Toc178150422)

[7.3 Radiotherapy 18](#_Toc178150423)

[7.3.1 Patient set-up and simulation 18](#_Toc178150424)

[7.3.2 Definition of target volume 18](#_Toc178150425)

[7.3.3 Definition of critical structures 19](#_Toc178150426)

[7.3.4 Dose prescription and normal tissue constraints 19](#_Toc178150427)

[7.3.5 Unplanned interruption of radiotherapy and treatment of radiotherapy-related side effects 20](#_Toc178150428)

[7.4 Nivolumab consolidation 20](#_Toc178150429)

[7.4.1 Doses and treatment regimens 20](#_Toc178150430)

[7.4.2 Management of toxicity 21](#_Toc178150431)

[Table 1: Recommended Dose Modifications for Nivolumab. 21](#_Toc178150432)

[7.5 Concomitant medications 28](#_Toc178150433)

[8. Schedule of Assessments 29](#_Toc178150434)

[9. Safety and Reporting 35](#_Toc178150435)

[9.1 Definition 35](#_Toc178150436)

[9.2 Adverse Event Collection and Reporting Information 36](#_Toc178150437)

[10. Central Imaging Review Committee 38](#_Toc178150438)

[11. Data management and record retention 38](#_Toc178150439)

[12. Data Monitoring Committee 39](#_Toc178150440)

[13. Statistical Analysis 39](#_Toc178150441)

[13.1 Sample size 39](#_Toc178150442)

[13.2 Statistical plan 39](#_Toc178150443)

[13.2.1 Description of analysis sets 39](#_Toc178150444)

[13.2.2 Methods of statistical analyses 40](#_Toc178150445)

[Reference List 41](#_Toc178150446)

# 1. Background

### 1.1 Overview of the treatment of locally advanced non-small cell lung cancer

The incidence of lung cancer is still increasing in most countries, and it remains the leading cause of cancer death ^1^. Non–small-cell lung cancer (NSCLC) accounts for approximately 80% to 85% of lung cancer. Most patients with NSCLC are detected with locally advanced or metastatic disease and have poor survival ^2^. Inoperable locally advanced NSCLC (LANSCLC) has shown an approximately 5% 5-year survival rate with radiation therapy (RT) alone. For the treatment of LA-NSCLC, multidisciplinary therapy has resulted in survival improvements. In the 1980s, induction chemotherapy was investigated for LA-NSCLC, and several randomized phase III studies of induction chemotherapy and subsequent RT have reported significant increases in survival ^3-5^. In the 1990s, a new approach with the concurrent administration of chemotherapy and RT was introduced and demonstrated further improvements in survival compared with RT alone ^6,7^. Subsequent randomized phase III trials have confirmed the superiority of concurrent chemoradiotherapy (CCRT) compared with sequential chemoradiotherapy^8,9^. Since then, the standard of care for LA-NSCLC has been CCRT. **Despite this progress with a combined-modality approach, the prognosis of LA-NSCLC remains poor, with a median survival time of 15 to 18 months**^10^.

### 1.2 Immunotherapy combined with radiotherapy

Immunotherapy has become standard of care in advanced NSCLC in a number of settings. The relative success of checkpoint inhibitors in NSCLC combined with the concept of ‘priming’ the immune system with radiation to make ‘cold’ less responsive tumours more responsive to immunotherapy, has opened the door to studies combining radiation with immunotherapy. Studies have shown that radiotherapy not only kills tumor cells directly, but also promotes the release of tumor antigens. The activation of antigen presenting cells (APC) and the combined immunotherapy further inhibits the depletion of T cells ^11,12^.

The main study investigating the efficacy of an anti-PD-1 axis inhibitor in association with radio-chemotherapy for locally advanced NSCLC was the PACIFIC trial^13,14^. This trial compared the PD-L1 inhibitor durvalumab vs. placebo in patients with locally advanced, unresectable stage III NSCLC who did not progress following concurrent platinum-based chemo-radiotherapy. It included 713 patients, who were randomized 2:1 to receive durvalumab 10 mg/kg every 2 weeks or placebo for up to 12 months. The co-primary endpoints were progression-free survival (PFS) and OS. Durvalumab was given until disease progression, other therapy initiation, severe collateral effects, or withdrawal of informed consent. The secondary endpoints were duration of response, objective response rate, percentage of patients alive without disease at 12 and 18 months, time to death or distant metastasis, quality of life, pharmacokinetics and immunogenicity. Median PFS resulted significantly higher for patients receiving Durvalumab than for patients receiving placebo (16.8 vs. 5.6 months respectively). The authors pointed out how the gain in PFS was achieved independently of PD-L1 expression (<25% vs. >25%). The median time to death or distant metastases was 23.2 months for durvalumab vs. 14.6 months for placebo, respectively, the objective response rate was 28.4% vs. 16%, respectively, and the median duration of response at 12 and 18 months was 72.8% vs. 56.1% and 46.8%, respectively. Patients who received durvalumab had a lower incidence of new metastases (20.4% vs. 32.1%) and in particular a lower incidence of brain metastases (5.5% vs. 11%).

The "concurrent chemoradiotherapy combined with pembrolizumab consolidation therapy for unresectable stage III NSCLC phase II study: the Hoosier cancer research network LUN 14-179 trial" reported on ASCO 2018 once again proved that pembrolizumab consolidation therapy after radiation chemotherapy significantly improved PFS. In this study, patients received Pembrolizumab (200 mg IV q3wk) for up to one year at 4-8 weeks after completion of radiotherapy and chemotherapy (carboplatin/paclitaxel, cisplatin/etoposide, or cisplatin/petromide +59-66.6 Gy XRT).The results of this study are very similar to those of PACIFIC. The median TMDD, PFS, 12- and 18-month PFS rates were very similar.

**According to the results of PACIFIC and lun14-179, immunotherapy is likely to be a good choice for consolidation treatment of locally advanced or advanced NSCLC patients after chemoradiotherapy.**

### 1.3 Advances in neoadjuvant immunotherapy for non-small cell lung cancer

Conventional NSCLC neoadjuvant chemotherapy can theoretically reduce tumor size, improve resectability of surgery, and eliminate or prevent micrometastases. However, the traditional neoadjuvant therapy also faces the risk of poor tumor regression and increased therapeutic toxicity. The sustained remission induced by PD1/pd-L1 inhibitors in some advanced NSCLC provides a possibility for its early application in NSCLC.

On April 16, 2018, the New England journal of Medicine (NEJM) published a study on the use of pd-1 antibody (nivolumab) as neoadjuvant therapy for non-small cell lung cancer (NSCLC), showing that two cycles of nivolumab before surgery may improve the outcome. Nine out of 20 cases (45%) presented with significant pathological responses to neoadjuvant nivolumab^15^. Furthermore, this study indicated the effect of immunotherapy on tumor micrometastasis in addition to the primary tumor. Of the 9 patients analyzed in this study, 8 had the same T cell clone amplification in peripheral blood and tumor specimens. A simple understanding of the results is that not only tumors generated a large numbers of tumor-specific T cells in response to nivolumab, but more importantly, tumor-specific T cells can enter the bloodstream and reach all parts of the body, including any potential micrometastatic sites. Since these T-cells are tumor-specific, they can remove these micrometastases, effectively reducing tumor recurrence and metastasis after surgery. Despite the high rate of major pathological response on histologic examination of the primary lung tumors, only two patients had a radiologic partial response. Two patients in whom tumors had increased in size on presurgical CT scans (although the increase was less than RECIST-defined progression) were found to have minimal or no residual tumor in the surgical specimen. These findings represent pathological evidence supporting the possibility that some patients may derive clinical benefit from immunotherapy without initial radiographic tumor shrinkage and that this process occurs because of immune-cell infiltration into the tumor, rather than true tumor growth.

**Based on the theoretical basis and clinical effect of pd-1 inhibitors in induction therapy for resectable NSCLC, we assume that the induction immunotherapy before RT of IIIA/B NSCLC can improve the survival of patients. Since immunotherapy cannot achieve significant reduction in tumor imaging in some patients, we will combine chemotherapy with pd-1 inhibitors in order to minimize tumor volume before radiotherapy, so as to reduce irradiation volume and improve the tolerance of subsequent radiotherapy**^16,17^**.**

### 1.4 Optimizing the Dose of Radiotherapy

The radiation dose and fractionation schedule are also important factors to consider when radiation is combined with immunotherapy. The conventional fractionation scheme, that is, 1.8 to 2-Gy fraction given once a day, takes advantage of tumors’ vulnerability in terms of DNA repair and cell cycle regulation. However, whether this conventional fractionation schedule, or one that utilizes a hypofractionated schedule (larger doses per day over a shorter course of time to a lower or same total dose) produces the best synergy with immunotherapy remains in question.

The PACIFIC trial indicates that local progression is the predominant pattern of treatment failure after CCRT and consolidative immunotherapy ^18^. This underscores the imperative to improve local control through optimized RT strategies. Previous studies have demonstrated that hypo-fractionated radiotherapy combined with concurrent chemotherapy (hypo-CCRT) offers superior local control in locally advanced NSCLC (LA-NSCLC) due to its higher biological equivalent dose (BED) compared to conventional fractionated RT^19^. Additionally, hypo-fractionated radiotherapy (hypo-RT) has shown a greater capacity to preserve peripheral lymphocytes by employing fewer dose fractions and smaller treatment fields^20,21^.

**In summary, radiotherapy combined with immunotherapy as a new combined treatment strategy is expected to further improve the survival of locally advanced NSCLC. Neoadjuvant immunotherapy has a certain theoretical basis, and has shown a preliminary good effect in operable NSCLC. From the results of PACIFIC and lun14-179 studies, immunotherapy is likely to be an excellent choice for consolidation treatment of locally advanced NSCLC patients after chemoradiotherapy. Therefore, we plan to conduct a phase II, randomized trial to explore the efficacy and safety of Nivolumab as consolidation therapy in patients with locally advanced, unresectable NSCLC who have not progressed following neoadjuvant chemotherapy plus Nivolumab and definitive concurrent chemoradiation.**

# 2. Study Objectives

| **Primary Objective** | **Outcome Measure** |
| --- | --- |
| To assess the efficacy of Nivolumab consolidation compared with observation in terms of PFS | PFS assessment according to RECIST 1.1 |

| **Secondary Objective** | **Outcome Measure** |
| --- | --- |
| To assess the efficacy of Nivolumab consolidation compared with observation in terms of OS and ORR | ORR assessment according to RECIST 1.1 |
| To assess the safety of Nivolumab consolidation compared with observation in terms of AE | AE assessment according to CTCAE 5.0 |
| To assess the symptoms and health-related quality of life in patients treated with Nivolumab consolidation compared with observation | EORTC QLQ-C30  LC13 |
| To assess the efficacy of neoadjuvant chemotherapy plus nivolumab in terms of ORR after neoadjuvant therapy | ORR assessment according to RECIST 1.1 |

| **Exploratory Objective** | **Outcome Measure** |
| --- | --- |
| To assess the efficacy of neoadjuvant chemotherapy plus nivolumab in terms of radiation dosimetric change after neoadjuvant therapy | Changes in tumor volume and lungs’ irradiated dose under a definitive radiation dose |
| To assess the efficacy of neoadjuvant chemotherapy plus nivolumab in terms of pulmonary function recovery after neoadjuvant therapy | Changes in forced expiratory volume in 1 second (FEV1) and pulmonary perfusion |
| To investigate the relationship between a patient’s PD-L1 expression and spatial distribution within the tumour microenvironment and efficacy outcomes | Tumoural expression of PD-L1 and spatial distribution within the tumour microenvironment relative to efficacy outcomes (OS, PFS and ORR) |
| To collect blood and tissue samples for analysis of peripheral and tumoural biomarkers | Biomarker analysis of blood and tissue to assess exploratory markers which may include but is not limited to: immune cell gene expression profiles within the peripheral and tumoural compartments, the presence of IFN-γ tumour necrosis factor-α, IL-2, IL-6, IL-10, IL-8, and IL-12 as well as antibodies against tumour, self, or viral antigens, expression of PD-L1 and the number and phenotype of immune cells such as T-cells |
| To collect stool samples for analysis of gut microorganisms | To assess the effect of nivolumab on gut microorganisms |

OS, Overall survival; PFS, Progression free survival; RECIST, Response Evaluation Criteria In Solid Tumours; EORTC QLQ-C30, European Organisation for Research and Treatment of Cancer 30-item core quality of life questionnaire; LC13, Lung Cancer Module; ORR, Objective response rate; ECOG PS, Eastern Cooperative Oncology Group performance status.

# 3. Ethical Considerations

The Institutional Review Board [IRB] should approve the final study protocol, including the final version of the Informed Consent Form and any other written information and/or materials to be provided to the patients. The Investigator will ensure the distribution of these documents to the applicable Ethics Committee, and to the study site staff. The opinion of the Ethics Committee should be given in writing. The Investigator should submit the written approval to Bristol-Myers Squibb before enrolment of any patient into the study.

The Ethics Committee should approve all advertising used to recruit patients for the study. Investigator should approve any modifications to the Informed Consent Form that are needed to meet local requirements. If required by local regulations, the protocol should be re-approved by the Ethics Committee annually.

The EC will assure that the study will be conducted in accordance with Good Clinical Practice (GCP), as defined by the International Conference on Harmonization (ICH), WHO and any local directives. The study will be conducted with personnel who are qualified by education, training, and experience to perform their respective tasks and that the study will not use the service of study personnel for whom sanctions have been invoked or where there has been scientific misconduct or fraud.

# 4. Informed Consent

The Investigator(s) at each center will:

- Ensure each patient is given full and adequate oral and written information about the nature, purpose, possible risk and benefit of the study;
- Ensure each patient is notified that they are free to discontinue from the study at any time.
- Ensure that each patient is given the opportunity to ask questions and allowed time to consider the information provided;
- Ensure each patient provides signed and dated informed consent before conducting any procedure specifically for the study;
- Ensure the original, signed Informed Consent Form(s) is/are stored in the Investigator’s Study File;
- Ensure a copy of the signed Informed Consent Form is given to the patient;
- Ensure that any incentives for patients who participate in the study as well as any provisions for patients harmed as a consequence of study participation are described in the informed consent form that is approved by an Ethics Committee.

The patient must meet the inclusion and exclusion criteria specified in Section“**Eligibility**”.

# 5. Study Design

## 5.1 Study design scheme

Patient screening and enrollment

Neoadjuvant chemotherapy and nivolumab

Nivolumab

Observation

Definitive radiotherapy, concurrent weekly DP

Patient screening and randomization

Follow up

1:1

Baseline assessment

2nd assessment

3rd assessment

**Figure 1: the study flowchart.**

This is a Phase II, randomized, multi-center study assessing the efficacy and safety of Nivolumab compared with observation, as sequential therapy in patients with locally advanced, unresectable NSCLC (Stage III), who have not progressed following neoadjuvant chemotherapy plus Nivolumab and definitive, platinum-based, concurrent chemoradiation therapy.

Approximately 264 patients will be enrolled and receive neoadjuvant chemotherapy plus Nivolumab. Approximately 224 (224/264, 85%) patients will receive definitive, concurrent chemoradiation (for patients who are suitable for definitive concurrent chemoradiation following neoadjuvant therapy). Approximately 168 (168/224, 75%) patients will be randomized (for patients in CR, PR or have SD following definitive, platinum-based, concurrent chemoradiation therapy), in a 1:1 ratio (Nivolumab to observation) to 1 of 2 arms: • Nivolumab (360 mg every 3 weeks [Q3W] intravenous [iv] for up to 12 months) • observation. Randomization will be stratified by: age at randomization (<65 versus ≥65 years of age), sex (male versus female), smoking history (smoker versus non-smoker) and EGFR status (wild type versus mutation type). Patients must complete their last dose of radiation therapy within 2 months prior to randomization in the study (the last dose of radiation therapy is defined as the day of the last radiation treatment session). For patients who are recovering from toxicities associated with prior treatment, randomization may be delayed by up to 4 months from the end of the chemoradiation therapy.

Tumor assessments will be performed using computed tomography/magnetic resonance imaging. The baseline assessment (before neoadjuvant therapy) should be performed within 28 days prior to enrollment. The second assessment should be performed 7 to 14 days after the last cycle of neoadjuvant therapy, and within 14 days before concurrent radiotherapy. The third assessment should be performed 2 months post the end of chemoradiation therapy, and ideally as close as possible before the start of nivolumab. Efficacy for all patients will be assessed by objective tumor assessments every 3 months (± 1 week) (relative to the date of randomization) for the first 36 months, then every 6 months (± 2 weeks) thereafter, until confirmed objective disease progression as defined by Response Evaluation Criteria In Solid Tumors (RECIST) 1.1 (irrespective of the reason for stopping nivolumab and/or subsequent therapy). If an unscheduled assessment is performed, and the patient has not progressed, every attempt should be made to perform the subsequent assessments at their scheduled visits.

Once a patient has had objective progression recorded and has discontinued nivolumab, the patient will be followed up for survival status every 3 months until death, withdrawal of consent or the end of the study.

## 5.2 Investigational products

### 5.2.1 Identification of investigational products

| Investigational product | Dosage form | manufacturer |
| --- | --- | --- |
| Opdivo (Nivolumab) | 100mg/10ml | Bristol-Myers Squibb |

### 5.2.2 Storage of investigational products and use conditions

**Nivolumab Injection**

Vials of nivolumab injection must be stored at 2℃ to 8℃ and protected from light and freezing. The unopened vials can be stored at room temperature (up to 25℃) and room light for up to 48 hours.

**Undiluted Nivolumab injection and diluted Nivolumab Injection in the IV Container**

The administration of nivolumab infusion must be completed with 24 hours of preparation. If not used immediately, the infusion solution may be stored under refrigeration conditions (2℃ to 8℃) for up to 24 hours, and a maximum of 8 hours of the total 24 hours can be at room temperature (up to 25℃) and room light. The maximum of 8 hours under room temperature and room light conditions includes the product administration period.

## 5.3 Patient enrolment

At the first screening (before neoadjuvant therapy), the Principal Investigator, or suitably trained delegate, will: 1. Obtain signed informed consent from the potential patient before any study specific procedures are performed. 2. Assign potential patient a unique enrolment number. Enrolment codes will start at 001 in each center and go up sequentially (eg, at Centre 01, patients will be assigned E codes E01001, E01002, etc.). This number is the patient’s unique identifier and is used to identify the patient on the electronic case report forms (eCRFs). 3. Determine patient eligibility. See Section “Eligibility”.

## 5.4 Multi-Disciplinary Discussion

Before enrolment, all patients will be discussed within the Multi-Disciplinary Team (MDT) and resectability assessed by 2 thoracic surgeons. Patients with stage IIIA disease will be judged as unresectable due to tumor invasion to mediastinum, vertebral body, bulky N2 disease, or R0 resection not achievable without pneumonectomy.

## 5.5 Procedures for Randomization

Patients must not be randomized unless all eligibility criteria have been met. At the second screening, patients who satisfy all the entry criteria will be centrally assigned to nivolumab or observation according to the randomization scheme generated by the biostatistics staff. Patients will be randomized in a 1:1 ratio to either Nivolumab consolidation or observation. Patients will be stratified at randomization based on their: age at randomization (<65 years versus ≥65 years of age), sex (male versus female), smoking history (smoker versus non-smoker) and EGFR status (wild type versus mutation type). The randomization scheme will be produced by a computer software program.

## 5.6 Duration of study

The duration of this study is expected to be 4 years. We expect 2 years to enroll patients and 2 years to follow up patients.

# 6. Eligibility

## 6. 1 Inclusion criteria

### For inclusion in neoadjuvant therapy, patients should fulfil the following criteria:

- Provision of signed, written and dated informed consent prior to any study specific procedures；
- Male or female aged 18 years to 75 years；
- Patients must have histologically- or cytologically-documented NSCLC who present with locally advanced, unresectable (Stage III) disease;
- Without prior chemotherapy, radiotherapy, surgery, targeted therapy or immunotherapy;
- Tumour sample requirements: Mandatory provision of an unstained, archived tumour tissue sample in a quantity sufficient to allow for analysis;
- A recent tumour biopsy (taken following completion of the most recent therapy) is an optional requirement, provided that a biopsy procedure is technically feasible and the procedure is not associated with unacceptable clinical risk;
- Life expectancy ≥12 weeks;
- World Health Organization (WHO) Performance Status of 0 or 1;
- Evidence of post-menopausal status, or negative urinary or serum pregnancy test for female pre-menopausal patients within 14 days before the use of study drug (HCG has a minimum sensitivity of 25 IU/L or equivalent);
- Women must be non-breastfeeding
- Women of reproductive age (WOCBP) must agree to comply with the contraceptive method during the study nivolumab treatment and for a period of 5 months following the last administration of the study treatment (i.e., 30 days [ovulation cycle] plus approximately 5 half-lives of the study drug).
- Men who have sex with WOCBP must agree to comply with the contraceptive method during the study nivolumab treatment and for 7 months after the last administration of the study treatment (i.e. 90 days [sperm renewal cycle] plus approximately 5 half-life of the study drug).
- Spermless men do not have to comply with contraceptive requirements. WOCBP who continues to be asexual with the opposite sex does not have to comply with contraceptive requirements, but must still undergo the pregnancy tests described in this section.
- Adequate organ and marrow function as defined below:
  - Forced expiratory volume in 1 second (FEV1) ≥800ml
  - Absolute neutrophil count >1.5 x 10^9^/L (1500 per mm3)
  - Platelets >100 x 10^9^/L (100,000 per mm3)
  - Haemoglobin≥9.0 g/dL (5.59 mmol/L)
  - Serum creatinine CL >50 mL/min by the Cockcroft-Gault formula (Cockcroft and Gault 1976)
  - Serum bilirubin ≤1.5 x upper limit of normal (ULN).
  - AST and ALT ≤2.5 x ULN

## 6.2 Exclusion criteria

### 6.2.1 Exclusion criteria for enrolment for neoadjuvant therapy

Patients should not enter the study if any of the following exclusion criteria are fulfilled:

- Concurrent enrolment in another clinical study, unless it is an observational(non-interventional) clinical study;
- Mixed small cell and non-small cell lung cancer histology;
- Current or prior use of immunosuppressive medication within 28 days before the first dose of Nivolumab, with the exceptions of intranasal and inhaled corticosteroids or systemic corticosteroids at physiological doses, which are not to exceed 10 mg/day of prednisone, or an equivalent corticosteroid. Systemic steroid administration required to manage toxicities arising from radiation therapy delivered as part of the chemoradiation therapy for locally advanced NSCLC is allowed.
- Prior exposure to any anti-PD-1 or anti-PD-L1 antibody;
- Recent major surgery within 4 weeks prior to entry into the study (excluding the placement of vascular access) that would prevent administration of nivolumab;
- Active or prior documented autoimmune disease within the past 2 years;
- Active or prior documented inflammatory bowel disease (eg, Crohn‘s disease, ulcerative colitis);
- History of primary immunodeficiency;
- History of organ transplant that requires therapeutic immunosuppression;
- Mean QT interval corrected for heart rate (QTc) ≥470 ms calculated from3 electrocardiograms (ECGs) using Bazett‘s Correction;
- Uncontrolled intercurrent illness including, but not limited to, ongoing or active infection, symptomatic congestive heart failure, uncontrolled hypertension, unstable angina pectoris, cardiac arrhythmia, active peptic ulcer disease or gastritis, active bleeding diatheses including any patient known to have hepatitis B, hepatitis C or human immunodeficiency virus (HIV), or psychiatric illness/social situations that would limit compliance with study requirements or compromise the ability of the patient to give written informed consent;
- Known history of tuberculosis, except for obsolete pulmonary tuberculosis;
- Receipt of live attenuated vaccination within 30 days prior to study entry or within30 days of receiving nivolumab;
- History of another primary malignancy within 5 years prior to starting nivolumab, except for adequately treated basal or squamous cell carcinoma of the skin or cancer of the cervix in situ and the disease under study;
- Female patients who are pregnant, breast-feeding or male or female patients of reproductive potential who are not employing an effective method of birth control;
- Any condition that, in the opinion of the investigator, would interfere with evaluation of the nivolumab or interpretation of patient safety or study results.

### 6.2.2 Exclusion criteria for concurrent chemoradiation following neoadjuvant therapy

Patients should not enter the concurrent chemoradiation phase if any of the following exclusion criteria are fulfilled:

- Patients who develop distant metastasis;
- Patients who develop locoregional disease progression, and the irradiation dose of normal tissue will exceed the limit as defined in Section 7.
- World Health Organization (WHO) Performance Status of 2-4;
- Inadequate organ and marrow function as defined below:
  - Forced expiratory volume in 1 second (FEV1) <800ml
  - Absolute neutrophil count <1.5 x 10^9^/L (1500 per mm3)
  - Platelets <100 x 10^9^/L (100,000 per mm3)
  - Haemoglobin<9.0 g/dL (5.59 mmol/L)
  - Serum creatinine CL <50 mL/min by the Cockcroft-Gault formula (Cockcroft and
- Gault 1976)
  - Serum bilirubin >1.5 x upper limit of normal (ULN).
  - AST and ALT >2.5 x ULN

### 6.2.3 Further exclusion criteria for randomization into Nivolumab consolidation or observation group

- Patients who have progressed whilst definitive platinum based, concurrent chemoradiation therapy;
- Current or prior use of immunosuppressive medication within 28 days before the first dose of Nivolumab, with the exceptions of intranasal and inhaled corticosteroids or systemic corticosteroids at physiological doses, which are not to exceed 10 mg/day of prednisone, or an equivalent corticosteroid. Systemic steroid administration required to manage toxicities arising from radiation therapy delivered as part of the chemoradiation therapy for locally advanced NSCLC is allowed.
- Any unresolved toxicity CTCAE >Grade 2 from the prior chemoradiation therapy will be excluded from randomization;
- Patients with Grade ≥2 pneumonitis from prior chemoradiation therapy will be excluded from randomization;
- Any prior Grade ≥3 immune-related adverse event (irAE) while receiving any previous immunotherapy agent, or any unresolved irAE>Grade 1.

## 6.3 Withdrawal from study

Patients may be discontinued from the study in the following situations:

- Patient decision. The patient is at any time free to discontinue treatment, without prejudice to further treatment
- Severe non-compliance to study protocol that, in the opinion of the investigator or sponsor, warrants withdrawal; eg, refusal to adhere to scheduled visits
- Patient lost to follow-up
- Pregnancy or intent to become pregnant.
- Adverse effects / lab abnormality or intercurrent illness that in opinion of investigator indicates that continued participation is nor in the best interest of the patient.

# 7. Treatment plan

## 7.1 Neoadjuvant therapy before radiotherapy

The neoadjuvant therapy before radiotherapy comprises of Docetaxel 60 mg/m^2^ d1+ Cisplatin 25 mg/m^2^ d1-3+Nivolumab 360 mg d1, Q3W, for a total of 2 cycles.

Nivolumab administration:

Nivolumab will be administered within 1 hour, prior to docetaxel and cisplatin, diluting with 100 mL 0.9% (weight/volume) saline.

Docetaxel administration:

Docetaxel will be diluted with 0.9% (weight/volume) saline, with final concentration not exceeding 0.9mg/mL, and administrated intravenously within 1 hour.

Cisplatin administration:

Cisplatin (25 mg/m^2^) will be diluted with 500 mL 0.9% (weight/volume) saline and administrated intravenously within 3 hours for 3 days.

## 7.2 Chemotherapy concurrent with radiotherapy

Docetaxel 25 mg/m^2^for 1 hour +Cisplatin 25 mg/m^2^, QW, for a total of 4 weeks.

## 7.3 Adverse effect of chemotherapy and modification

Dose-limiting toxicity of docetaxel is neutropenia. Other possible adverse events include allergies, skin reactions, gastrointestinal toxicity (nausea, vomiting, stomatitis, and diarrhea), hair loss, muscle weakness, mild injection site reaction (phlebitis), peripheral neuropathy, and fluid retention/edema. Major adverse effects of cisplatin monotherapy include ototoxicity, peripheral neuropathy, renal failure, and vomiting.

All patients are given the scheduled chemotherapy dose, which could be adjusted for the most severe hematology or other toxicity if necessary. Any patient requiring a reduced dose will continue to receive the reduced dose throughout the subsequent treatment cycle. If multiple toxicities occur in the patient and the dose adjustment principles differ from one another, the minimum dose is selected. Any patient who has had two times of dose reductions (for the same drug) should discontinue chemotherapy when a third dose reduction is required due to toxicity. Chemotherapy can be delayed for up to 2 weeks, or it needs to be canceled.

**Modification for hematology toxicities**

**The dose of docetaxel and cisplatin should be decreased by 25%, if any of the following events occurred in the previous cycle:**

·Absolute neutrophil count < 0.5*10^9/L;

·Neutropenia with infection;

·Platelets <25 *10^9/L.

**The dose of docetaxel and cisplatin should be decreased by 50%, if any of the following events occurred in the previous cycle:**

·Neutropenia with a fever ≥ 38.5 degrees

·Platelets <25 *10^9/L with bleeding.

| ANC (absolute neutrophil count)≥ 1.5×10^9/Land platelet ≥100×10^9/L | Full dose |
| --- | --- |
| ANC <1.5×10^9/L or platelet≥100×10^9/L | Hold chemotherapy for up to 2 weeks;  Resume full-dose chemotherapy when ANC ≥ 1.5×10^9/L and platelet ≥100×10^9/L |
| Febrile neutropenia | Hold chemotherapy for up to 2 weeks;  Resume chemotherapy with a dose reduced by 25% when ANC ≥ 1.5×10^9/L and platelet ≥100×10^9/L; |

**Modification for renal toxicities**

| Calculated CCr≥ 50 ml/min | Full dose |
| --- | --- |
| Calculated CCr≥ 50 ml/min | Continue docetaxel，hold cisplatin; Restart full dose cisplatin when CCr≥ 50 ml/min. |

**Modification for neuropathy**

| Grade 3 or 4 | Hold docetaxel and cisplatin |
| --- | --- |
| Grade 2 | Reduce dose by 25% |
| Grade 1 | Full dose |

**Modification for stomatitis**

| Grade 3 or 4 | Continue cisplatin, hold docetaxel.  Restart docetaxel with a dose reduced by 25% when resolve to grade≤2. |
| --- | --- |

**Modification for Allergy**

| Grade 1：  Local skin reactions such as mild itching, flush and rash | - Consider slowing down the infusion until symptoms resolve; - Bedside observation and monitoring; - Then finish the scheduled dose of docetaxel. |
| --- | --- |
| Grade 2：  Symptoms other than grade 1or 3-4, including any of the following: systemic pruritus, flushing and rash; dyspnea; hypotension with systolic blood pressure > 80 mm Hg | - Hold the infusion of docetaxel - Diphenhydramine 50 mg IV with or without dexamethasone 10 mg IV; - Monitor the patient until symptoms resolve - Restart docetaxel infusion after symptoms disappear. The docetaxel infusion should be restarted at a slow rate and then gradually increased to normal rate. - For the next cycle, give antihistamines in advance, start the infusion with a slow rate and gradually speed up to normal rate. |
| Grade 3-4：  Any of the following: bronchospasm, systemic rubella, systolic blood pressure <80mm Hg, vascular edema. | - Discontinue the infusion of docetaxel immediately; - Diphenhydramine 50 mg IV, with or without dexamethasone 10 mg IV, and/or epinephrine as required; monitor the patient until symptoms resolve. |

**Modification for other non-hematologic toxicities：**

For grade≤2 AE, if possible, do not reduce the dose of chemotherapy. For grade≥3 AE, docetaxel and cisplatin should be held until recovery to grade 1 or less, and then retreated with a reduced dose by 25% for both drugs. The delay should not exceed two weeks.

## 7.3 Radiotherapy

Response to neoadjuvant therapy is evaluated within 7-14 days after neoadjuvant therapy, and radiation therapy is initiated 21 to 28 days after the second cycle of neoadjuvant therapy in patients without distant metastasis.

Prior to radiotherapy, radiation oncologists should assess the patient’s chest CT to ensure that the dose constraints of specific normal tissues will be met at a definitive radiation dose.

### 7.3.1 Patient set-up and simulation

Individual patient is immobilized in a supine position and scanned from the Atlas (C1) to the second lumbar vertebra (L2) level. Simulation four-dimensional CT (4DCT) scanning is performed using helical CT, a 3-mm slice thickness and intravenous contrast, to account for tumor motion. The simulation images are transferred to the planning system (Monaco; Elekta Medical Systems). IV contrast is encouraged for better delineation between tumor, atelectasis, and vascular structures as well as better definition of normal tissue contours.

### 7.3.2 Definition of target volume

Gross tumor volume (GTV) is defined as visible primary tumors (GTV-T) and involved lymph nodes (GTV-N) on CT and/or PET scans. The GTVs are composite volumes from CT scans of all breathing phases. The criteria of lymph node positivity include: short axis size ≥ 10 mm on pretreatment CT scan, reported positive on the pretreatment PET scan, or biopsy positive on mediastinoscopy or endobronchial ultrasound-guided biopsies. Clinical target volume (CTV) includes the primary lung tumor with a 0.6 cm margin, the ipsilateral hilum and involved lymph nodes region. The lymph nodal regions are delineated according to the new lymph node map of the International Association for the Study of Lung Cancer (IASLC). The planning target volume for GTV (PTV_GTV) covers the GTV-T and GTV-N with a 0.5-cm margin. PTV_CTV covers the CTV with a 0.5-cm margin.

### 7.3.3 Definition of critical structures

Critical structure contours will be drawn in axial planes of the primary planning dataset. In general, critical structures should be contoured if they are found within an axial slice within 3 cm in the craniocaudal direction of any PTV slice treated on protocol.

Spinal Cord The spinal cord will be contoured based on the bony limits of the spinal canal. The spinal cord should be contoured starting at least 3 cm above the superior extent of any PTV and continuing on every CT slice to at least 3 cm below the inferior extent of any PTV.

Esophagus The esophagus will be contoured using mediastinal windowing on CT to correspond to the mucosal, submucosa, and all muscular layers out to the fatty adventitia. The esophagus should be contoured starting at the orifice to the gastroesophageal junction.

Heart The heart will be contoured along with the pericardial sac. The superior aspect (or base) for purposes of contouring will begin at the level of the inferior aspect of the aortic arch (aortopulmonary window) and extend inferiorly to the apex of the heart.

Whole Lung Both the right and left lungs should be contoured individually (Lung_L, Lung_R) and also combined as one structure (Lungs). Contouring should be carried out using pulmonary windows. All inflated and collapsed lung should be contoured; however, gross tumor (GTV) and trachea/ipsilateral bronchus as defined above should not be included for the structure created and -labeled as Lungs – GTV.

Proximal bronchial tree Proximal bronchial tree includes the distal 2 cm of the trachea, the carina, the right and left mainstem bronchi, the right and left upper lobe bronchi, the bronchus intermedius, the right middle lobe bronchus, the lingular bronchus, and the right and left lower lobe bronchi.

### 7.3.4 Dose prescription and normal tissue constraints

Thoracic radiotherapy is administered using a split-course hypo-fractionated CCRT (hypo-CCRT) scheme.

Patients initially receive the first course of hypo-CCRT. Subsequently, a mid-treatment evaluation including chest/upper abdominal contrast-enhanced CT and pulmonary function test is performed 2-3 weeks after the completion of the first course to assess tumor response and patients’ physical conditions. Patients who do not experience disease progression or persistent ≥ grade 2 (G2+) toxicities are eligible to receive the hypo-CCRT-boost course 3-4 weeks after the completion of the first course, in which an adaptive radiotherapy plan is applied to target the residual tumor. For those who do not recover from G2+ toxicities at mid-treatment evaluation, a re-evaluation for the boost course in another 2 weeks is permitted.

The dose-fraction regimens include a total dose of 40Gy in 10 daily fractions in the first course followed by 24Gy in 6 daily fractions in the boost course, or a total dose of 30Gy in 6 daily fractions in the first course followed by 24~30Gy in 6 daily fractions in the boost course.

**Normal Structure Constraints**

| **Organs at risk** | **Dose constraints** | | | |
| --- | --- | --- | --- | --- |
|  | **Dose regimen 1** | | **Dose regimen 2** | |
|  | The first course  (40Gy/10Fr) | The boost course  (24Gy/6Fr) | The first course  (30Gy/6Fr) | The boost course  (24~30Gy/6Fr) |
| Lungs | V15≤20% | V10≤10% | V15≤10% | V15≤10% |
| Ipsilateral lung | V15≤25% | V10≤15% | V15≤20% | V15≤20% |
| Spinal cord | Dmax≤20Gy | Dmax≤10Gy | Dmax≤12Gy | Dmax≤12Gy |
| Heart | V20≤20% | V10≤10% | V20≤15% | V20≤15% |
| Esophagus | Dmax≤36Gy,  V30≤20% | Dmax≤20Gy,  V10≤20% | Dmax≤25Gy,  V20≤20% | Dmax≤24Gy,  V20≤20% |
| Great vessels | Dmax≤44Gy,  V40≤10cc | Dmax≤22Gy,  V20≤10cc | Dmax≤33Gy,  V30≤10cc | Dmax≤33Gy,  V30≤10cc |
| Proximal bronchial tree | Dmax≤44Gy | Dmax≤22Gy | Dmax≤33Gy | Dmax≤26.4Gy |

### 7.3.5 Unplanned interruption of radiotherapy and treatment of radiotherapy-related side effects

Unplanned radiotherapy interruptions should be avoided as far as possible. If radiotherapy is interrupted due to treatment-related side effects, concurrent chemotherapy should be interrupted accordingly. Treatment may continue after the side effects have recovered below grade 2.Radiotherapy should not be interrupted for more than 2 weeks. In order to ensure the implementation of radiotherapy, corresponding preventive measures and nutritional support treatment can be adopted.

Specifically, treatment of radiation pneumonitis and esophagitis is detailed below:

|  | Side effects  NCI CUCAE 4.0 | Treatment |
| --- | --- | --- |
| Radiation esophagitis | Grade 1 | None |
|  | Grade 2 | Supportive treatment |
|  | Grade 3 | Lidocaine gel + H2 receptor inhibitor + sucralfate, and/or hold radiotherapy;  Resume RT If recover to ≤Grade 2. |
|  | Grade 4 | H2 receptor inhibitor + PPI， and/or hold RT;  Resume radiotherapy If recover to ≤Grade 2. |
| Radiation pneumonits | Grade 1 | Prevention of infection, smoking patients are strongly recommended to stop smoking during radiotherapy. |
|  | Grade 2 | Oxygen supply, nutritional support |
|  | Grade 3 | Hold radiotherapy, antibiotics,  Resume radiotherapy If recover to ≤Grade 2. |
|  | Grade 4 | Discontinue radiotherapy permanently,  Prednisone and antibiotics |

## 7.4 Nivolumab consolidation

### 7.4.1 Doses and treatment regimens

Patients will receive either Nivolumab consolidation (360 mg) via iv infusion Q3W±3 days or observation after chemo-radiotherapy. Administration of nivolumab will commence within 3 days following randomization to Nivolumab after confirmation of eligibility and will continue on a Q3W schedule for a maximum duration of 12 months. Nivolumab should be discontinued prior to 12 months if there is confirmed PD (unless the investigator considers the patient continues to receive benefit from nivolumab), initiation of alternative cancer therapy, unacceptable toxicity, withdrawal of consent, or other reasons to discontinue nivolumab occur.

Doses of 360mg will be administered using a 100 mL iv bag containing 0.9% (weight/volume) saline and delivered through an iv administration for 1 hour.

Patients who achieve and maintain disease control (CR, PR, NED or SD) through to the end of the 12-month treatment period will enter follow-up per Table 3.

Patients who have a dose interruption due to toxicity at any point in the 12 months of treatment may resume and complete the 12-month treatment period.

Patients who have confirmed PD during the 12-month treatment period, and cannot continue to receive nivolumab will enter follow-up with assessments as shown in Table 5.

### 7.4.2 Management of toxicity

The following general guidance should be followed for management of toxicities.

1) Treat each of the toxicities with maximum supportive care.

2) If the symptoms promptly resolve with supportive care, consideration should be given to Nivolumab along with appropriate continuing supportive care. If medically appropriate, dose modifications are permitted (see below).

3) All dose modifications should be documented with clear reasoning and documentation of the approach taken.

In addition, there are certain circumstances in which nivolumab should be permanently discontinued (see Table 1). Following the first infusion of Nivolumab, subsequent administration can be modified based on toxicities observed as described in Table 1. All toxicities will be graded according to CTCAE Version 4.0. Dose reductions are not permitted. Dose modifications will not be required for Aes that are clearly not attributed to nivolumab (such as an accident) or for laboratory abnormalities that are not deemed to be clinically significant.

Based on the mechanism of action of Nivolumab leading to T-cell activation and proliferation, there is the possibility of observing irAEs during the conduct of this study. Potential irAEs may include immune-mediated enterocolitis, dermatitis, hepatitis, and endocrinopathies. Patients should be monitored for signs and symptoms of irAEs. In the absence of an alternate aetiology, (eg, infection or PD) signs or symptoms of enterocolitis, dermatitis, hepatitis, and endocrinopathy should be considered to be immune-related.

### Table 1: Recommended Dose Modifications for Nivolumab.

| **Adverse Reaction** | **Severity*** | **Dose Modification** |
| --- | --- | --- |
| Colitis | Grade 2 or 3 diarrhea or colitis | Withhold dose until resolution to ≤Grade 1. |
|  | Grade 4 diarrhea or colitis | Permanently discontinue |
| Pneumonitis | Grade 2 pneumonitis | Withhold dose until resolution to ≤Grade 1. If resolution to ≤ Grade 1 occurs within 3 days of the initiation of maximal supportive care (including corticosteroids), resume Nivolumab administration at the next scheduled dose. Otherwise, discontinue Nivolumab. |
|  | Grade 3 or 4 pneumonitis | Permanently discontinue |
| Hepatitis | Aspartate aminotransferase (AST) or alanine aminotransferase (ALT) more than 3 and up to 5 times the upper limit of normal (ULN) or total bilirubin more than 1.5 and up to 3 times the ULN. | Withhold dose until resolution to ≤Grade 1. |
|  | AST or ALT more than 5 times the ULN or total bilirubin more than 3 times the ULN | Permanently discontinue |
| Hypophysitis | Grade 2 or 3 hypophysitis | Withhold dose until resolution to ≤Grade 1. |
|  | Grade 4 hypophysitis | Permanently discontinue |
| Adrenal Insufficiency | Grade 2 adrenal insufficiency | Withhold dose until resolution to ≤Grade 1. |
|  | Grade 3 or 4 adrenal insufficiency | Permanently discontinue |
| Type 1 Diabetes Mellitus | Grade 3 hyperglycemia | Withhold dose until resolution to ≤Grade 1. |
|  | Grade 4 hyperglycemia | Permanently discontinue |
| Nephritis and Renal Dysfunction | Serum creatinine more than 1.5 and up to 6 times the ULN | Withhold dose until resolution to ≤Grade 1. |
|  | Serum creatinine more than 6 times the ULN | Permanently discontinue |
| Skin | Grade 3 rash or suspected Stevens-Johnson syndrome (SJS) or toxic epidermal necrolysis (TEN) | Withhold dose until resolution to ≤Grade 1. |
|  | Grade 4 rash or confirmed SJS or TEN | Permanently discontinue |
| Encephalitis | New-onset moderate or severe neurologic signs or symptoms | Withhold dose until resolution to ≤Grade 1. |
|  | Immune-mediated encephalitis | Permanently discontinue |
| Other | Other Grade 3 adverse reaction First occurrence | Withhold dose until resolution to ≤Grade 1. |
|  | Recurrence of same Grade 3 adverse reactions | Permanently discontinue |
|  | Life-threatening or Grade 4 adverse reaction | Permanently discontinue |
|  | Grade 3 myocarditis | Permanently discontinue |
|  | Requirement for 10 mg per day or greater prednisone or equivalent for more than 12 weeks | Permanently discontinue |
|  | Persistent Grade 2 or 3 adverse reactions lasting 12 weeks or longer | Permanently discontinue |

* Toxicity is graded per National Cancer Institute Common Terminology Criteria for Adverse Events. Version 5.0 (NCI CTCAE v5).

In addition to the dose modifications shown in **Table 1**, it is recommended the management guidelines for irAEs are followed, which are presented in **Table 2.**

**Table 2 Management and follow-up for immune-related adverse events.**

| Adverse effects | Grade | Management | Follow-up |
| --- | --- | --- | --- |
| Pneumonitis | 1 Radiographic changes only | Consider delay of I-O therapy;  Monitor for symptoms every 2-3 days;  Consider Pulmonary and ID consults. | Re-image at least every 3 weeks;  If worsens: Treat as Grade 2 or 3-4. |
|  | 2 Mild to moderate new symptoms | Delay I-O therapy per protocol;  Pulmonary and ID consults;  Monitor symptoms daily, consider hospitalization, 1.0mg/kg/day methylprednisolone IV or oral equivalent;  Consider bronchoscopy, lung biopsy | Re-image every 1-3 days;  If improves: When symptoms return to near baseline, taper steroids over at least 1 month and then resume I-O therapy per protocol and consider prophylactic antibiotics;  If not improving after 2 weeks or worsening: Treat as Grade3-4. |
|  | 3-4 Severe new symptoms; New/worsening hypoxia;  Life-threatening | Discontinue I-O therapy per protocol; Hospitalize;  Pulmonary and ID consults;  2-4 mg/kg/day methylprednisolone IV or IV equivalent;  Add prophylactic antibiotics for opportunistic infections;  Consider bronchoscopy, lung biopsy. | If improves to baseline: Taper steroids over at least 6 weeks;  If not improving after 48 hours or worsening: Add additional immunosuppression. |
| Hepatic | 1 AST or ALT > ULN to 3.0 x ULN and/or T. bili >ULN to1.5 x ULN | Continue I-O therapy per protocol; | monitoring per protocol;  If worsens:  Treat as Grade 2 or 3-4. |
|  | 2 AST or ALT > 3.0 to≤ 5 x ULN and/or T. bili >1.5 to≤3xULN | Delay I-O therapy per protocol;  Increase frequency of monitoring to every 3 days. | If returns to baseline:  Resume routine monitoring, resume I-O therapy per protocol;  If elevations persist > 5-7 days or worsen:  0.5-1mg/kg/day methylprednisolone or oral equivalent and when LFT returns to grade1or baseline, taper steroids over at least 1 month, consider prophylactic antibiotics for opportunistic infections, and resume I-O therapy per protocol. |
|  | 3-4 AST or ALT > 5xULN or T.bili >3xULN | Discontinue immunotherapy;  Increase frequency of monitoring to every 1-2 days;   - 1. to 2.0mg/kg/day methylprednisolone IV or IV equivalent;   Add prophylactic antibiotics for opportunistic infections;  Consult gastroenterologist. | If returns to grade 2:  Taper steroids over at least 1 month;  If does not improve in >3-5 days, worsens or rebounds:  Add mycophenolatemofetil 1g BID;  If no response within an additional 3-5 days, consider other immune suppressants per local guidelines. |
| Endocrinopathy | Asymptomatic TSH elevation | Continue immunotherapy per protocol;  Consider endocrinology consult. | If TSH < 0.5xLLN, or TSH>2xULN, or c onsistently out of range in 2 subsequent measurements: include fT4 at subsequent cycles as clinically indicated. |
|  | Symptomatic endocrinopathy | Evaluate endocrine function;  Consider pituitary scan;  Symptomatic with abnormal lab/pituitary scan: Delay immunotherapy per protocol;  1-2mg/kg/daymethylprednisolone IV or PO equivalent;  Initiate appropriate hormonetherapy.  No abnormal lab/pituitary MRI scan but symptoms persist: Repeat labs in 1-3weeks/ MRI in 1 month. | If improves (with or without hormone replacement):  Taper steroids over at least 1 month and consider prophylactic antibiotics for opportunistic infections;  Resume immunotherapy per protocol;  Patients with adrenal insufficiency may need to continue steroids with mineralocorticoid component. |
|  | Suspicion of adrenal crisis (e.g. severe dehydration, hypotension, shock out of proportion to current illness | Delay or discontinue immunotherapy per protocol;  Rule out sepsis;  Stress dose of IV steroids with mineralocorticoid activity;  IV fluids;  Consult endocrinologist;  If adrenal crisis ruled out, then treat as above for symptomatic endocrinopathy. |  |
| Rash | 1-2 Covering ≤30% BSA | Symptomatic therapy (e.g. antihistamines, topical steroids);  Continue immunotherapy per protocol. | If persists > 1-2 weeks or recurs:  Consider skin biopsy;  Delay immunotherapy per protocol;  Consider 0.5-1.0mg/kg/day methylprednisolone IV or oral equivalent.  Once improving, taper steroids over at least 1 month, consider prophylactic antibiotics for opportunistic infections, and resume immunotherapy per protocol  If worsens:  Treat as Grade3-4. |
|  | 3-4 Covering >30% BSA; Life threatening consequences | Delay or discontinue immunotherapy per protocol;  Consider skin biopsy;  Dermatology consult;  1.0-2.0 mg/kg/day IV methylprednisolone or IV equivalent | If improves to Grade1:  Taper steroids over at least 1 month and add prophylactic antibiotics for opportunistic infections;  Resume immunotherapy per protocol. |
| Neurological Toxicity | 1 Asymptomatic or mild symptoms; Intervention not indicated | Continue immunotherapy per protocol | Continue to monitor the patient.  If worsens:  Treat as Grade 2 or 3-4. |
|  | 2 Moderate symptoms; Limiting instrumental ADL | Delay immunotherapy per protocol;  Treat symptoms per local guidelines;  Consider 0.5 to 1.0mg/kg/day methylprednisolone IV or PO equivalent. | If improves to baseline:  Resume immunotherapy per protocol when improved to baseline.    If worsens:  Treat as Grade3-4. |
|  | 3-4 Severe symptoms; Limiting self-care ADL; Life-threatening | Discontinue immunotherapy per protocol;  Obtain neurology consult;  Treat symptoms per local guidelines;  1.0-2.0 mg/kg/day IV methylprednisolone or IV equivalent;  Add prophylactic antibiotics for opportunistic infections. | If improves to Grade2:  Taper steroids over at least 1month;  If worsens or atypical presentation: Consider IVIG or other immunosuppressive therapies per local guidelines. |

## 7.5 Concomitant medications

Investigators may prescribe concomitant medications or treatments (eg, acetaminophen, diphenhydramine) deemed necessary to provide adequate prophylactic or supportive care except for those medications identified as “excluded” as listed below: • Any chemotherapy, radiotherapy, immunotherapy, biologic, or hormonal therapy for cancer treatment concurrent with Nivolumab. Concurrent use of hormones for non cancer-related conditions (eg, insulin for diabetes and hormone replacement therapy) is acceptable.

• Immunosuppressive medications including, but not limited to systemic corticosteroids at doses beyond 10 mg/day of prednisone or equivalent, methotrexate, azathioprine, and tumour necrosis factor alpha blockers. Use of immunosuppressive medications for the prophylactic care for docetaxel or management of study drug-related AEs in patients is acceptable. In addition, use of inhaled and intranasal corticosteroids is permitted.

• Live attenuated vaccines within 30 days of dosing. Inactivated viruses such as those in the influenza vaccine are permitted.

Other medication, which is considered necessary for the patient’s safety and well-being, may be given at the discretion of the investigator and recorded in the appropriate sections of the eCRF.

# 8. Schedule of Assessments

The schedule for assessments at screening and during the Treatment Period is presented in Table 3. The schedule of study procedures during follow-up for patients who have completed study drug and achieved disease control (until confirmed PD) and patients who have discontinued study drug due to toxicity or a reason other than confirmed PD is presented in Table 4. The schedule of study procedures during follow-up for patients who have discontinued study drug due to confirmed PD is presented in Table 5.

**Table 3. The schedule for assessments at screening and during the Treatment Period**

| Assessment | | Before enrollment | During neoadjuvant therapy | During CCRT | Consolidation immunotherapy |
| --- | --- | --- | --- | --- | --- |
| Written informed consent | | Within 14 days |  |  |  |
| Randomisation | |  |  | 2 months post the end of CCRT |  |
| Medical history | -The diagnosis of lung cancer  -Concomitant medications/procedures  - Allergies  -General medical history (including comorbidities)  - Previous anti-cancer therapy | Within 14 days |  |  |  |
| Physical examination (PE) | -Vital Signs  -Height  -Weight  -ECOG. | Within 14 days | Every 3 weeks | Weekly | Every 3 weeks |
| Hematology | -Hemoglobin  -Platelet  -Leukocyte  -Neutrophil | Within 14 days | Every 3 days | Weekly | Every 3 weeks |
| Serum chemistry | - Liver and kidney function  -Electrolytes | Within 14 days | Weekly | Weekly | Every 3 weeks |
| Coagulation parameters | -Prothrombin time  -APTT  -INR | Within 14 days |  |  |  |
| Thyroid function tests | -TSH  -T3  -T4 | Within 14 days | Every 3 weeks | Pre | Every 3 weeks |
| Urinalysis |  | Within 14 days | Every 3 weeks | Every 2 weeks | Every 3 weeks |
| Pregnancy test | Urine hCG or serum βhCG | Within 14 days |  | If necessary | If necessary |
| Circulating soluble factors | Cytokines, chemokines, growth factors and antibodies against tumour and self antigens in circulation | Within 14 days | 7-14 days post | 2 months post the end of CCRT | Every 3 months |
| Tumor assessment | -Chest CT and CTA  -Abdominal CT  -Brain CT or MRI  -Bone scan (can be replaced by PET)  -Other necessary imaging | Within 28 days | 7-14 days post | 2 months post the end of CCRT | Every 3 months |
| Radation dosimetric assessment | -Tumor volume  -lungs’ V20, mean lung dose | Within 21 days | 7-14 days post |  |  |
| Electrocardiogram | Standard twelve leads electrocardiography | Within 21 days | Every 3 weeks | As clinically indicated | As clinically indicated |
| Lung function | PFT, pulmonary perfusion imaging | Within 21 days | 7-14 days Post | Mid-treatment and 2 months Post |  |
| Pathological examination | -Biopsy through bronchscopy, transthoracic needle pneumocentesis or mediastinoscopy  -PD-L1expression  -tumor mutation burden | Within 28 days |  |  |  |
| Others | Base on clinical needs (e.g. threshold audiogram) | Within 21 days |  | If necessary | If necessary |
| The assessment of AEs | CTC-AE 5.0 | Anytime |  | Anytime | Anytime |

**Table 4. The schedule of study procedures during follow-up for patients who have completed study drug and achieved disease control (until confirmed PD) and patients who have discontinued study drug due to toxicity or a reason other than confirmed PD.**

| Assessment | | Time since the last dose of Nivolumab |
| --- | --- | --- |
| Physical examination (PE) | -Vital Signs  -Height  -Weight  -ECOG | 1 month after the last dose |
| Hematology | -Hemoglobin  -Platelet  -Leukocyte  -Neutrophil | Once a month for four months |
| Serum chemistry | - Liver and kidney function  -Electrolytes | Once a month for two months |
| Coagulation parameters | -Prothrombin time  -APTT  -INR | 1 month after the last dose |
| Thyroid function tests | -TSH  -T3  -T4 | 1 month after the last dose |
| Urinalysis |  | 1 month after the last dose |
| Circulating soluble factors | Cytokines, chemokines, growth factors and antibodies against tumour and self antigens in circulation | 3 months after the last dose |
| Electrocardiogram |  | 1 month after the last dose |
| Tumor assessment | -Chest CT  -Abdominal CT  -Brain CT or MRI  -Other necessary imaging | Every 3 months relative to the date of randomization until confirmed PD by RECIST 1.1 by investigational site review. |
| Survival status: phone contact with patients who refuse to return for evaluations and agree to be contacted |  | Once a month for 6 months, every two months thereafter |
| The assessment of AEs | CTC-AE 5.0 | Once a month for three months after the last dose |

**Table 5. The schedule of study procedures during follow-up for patients who have discontinued study drug due to confirmed PD .**

| Assessment | | Time since the last dose of Nivolumab |
| --- | --- | --- |
| Physical examination (PE) | -Vital Signs  -Height  -Weight  -ECOG | 1 month after the last dose |
| Hematology | -Hemoglobin  -Platelet  -Leukocyte  -Neutrophil | Once a month for four months |
| Serum chemistry | - Liver and kidney function  -Electrolytes | Once a month for two months |
| Coagulation parameters | -Prothrombin time  -APTT  -INR | 1 month after the last dose |
| Thyroid function tests | -TSH  -T3  -T4 | 1 month after the last dose |
| Urinalysis |  | 1 month after the last dose |
| Circulating soluble factors | Cytokines, chemokines, growth factors and antibodies against tumour and self antigens in circulation | 1 month after the last dose |
| Electrocardiogram |  | 1 month after the last dose |
| Tumor assessment | -Chest CT  -Upper abdominal CT  -Brain CT or MRI  -Other necessary imaging | For patients who discontinue study drug following confirmed progression, scans should be conducted according to local clinical practice and submitted for central review until a new treatment is started (these scans are optional). |
| Survival status: phone contact with patients who refuse to return for evaluations and agree to be contacted |  | Once a month for 6 months, every two months thereafter |
| The assessment of AEs | CTC-AE 5.0 | Once a month for three months after the last dose, or till the initiation of new anti-cancer treatment, whichever comes first |

# 9. Safety and Reporting

## 9.1 Definition

**ADVERSE EVENTS**

An Adverse Event (AE) is defined as any new untoward medical occurrence or worsening of a preexisting medical condition in a clinical investigation participant administered study drug and that does not necessarily have a causal relationship with this treatment. An AE can therefore be any unfavorable and unintended sign (such as an abnormal laboratory finding), symptom, or disease temporally associated with the use of investigational product, whether or not considered related to the investigational product.

**SERIOUS ADVERSE EVENTS**

A **S*erious Adverse Event (SAE)*** is any untoward medical occurrence that at any dose:

- results in death
- is life-threatening (defined as an event in which the participant was at risk of death at the time of the event; it does not refer to an event which hypothetically might have caused death if it were more severe)
- requires inpatient hospitalization or causes prolongation of existing hospitalization (see **NOTE** below)
- results in persistent or significant disability/incapacity
- is a congenital anomaly/birth defect
- is an important medical event (defined as a medical event(s) that may not be immediately life-threatening or result in death or hospitalization but, based upon appropriate medical and scientific judgment, may jeopardize the subject or may require intervention [eg, medical, surgical] to prevent one of the other serious outcomes listed in the definition above.) Examples of such events include, but are not limited to, intensive treatment in an emergency room or at home for allergic bronchospasm; blood dyscrasias or convulsions that do not result in hospitalization.)
- Suspected transmission of an infectious agent (eg, pathogenic or nonpathogenic) via the study drug is an SAE.

Although pregnancy and potential drug-induced liver injury (DILI), are not always serious by regulatory definition, however, these events must be reported within the SAEs timeline.

Any component of a study endpoint that is considered related to study therapy should be reported as an SAE (eg, death is an endpoint, if death occurred due to anaphylaxis, anaphylaxis must be reported).

**NOTE**:

The following hospitalizations are not considered SAEs:

- - a visit to the emergency room or other hospital department < 24 hours, that does not result in admission (unless considered an important medical or life-threatening event)
  - elective surgery, planned prior to signing consent
  - admissions as per protocol for a planned medical/surgical procedure
  - routine health assessment requiring admission for baseline/trending of health status (eg, routine colonoscopy)
  - Medical/surgical admission other than to remedy ill health and planned prior to entry into the study. Appropriate documentation is required in these cases.
  - Admission encountered for another life circumstance that carries no bearing on health status and requires no medical/surgical intervention (eg, lack of housing, economic inadequacy, caregiver respite, family circumstances, administrative reason).
  - Admission for administration of anticancer therapy in the absence of any other SAEs (applies to oncology protocols)

## 9.2 Adverse Event Collection and Reporting Information

- All Serious Adverse Events (SAEs) that occur following the subject’s written consent to participate in the study through 100 days of discontinuation of dosing must be reported to BMS Worldwide Safety, whether related or not related to study drug. If applicable, SAEs must be collected that relate to any later protocol-specified procedure (eg, a follow-up skin biopsy).
- Following the subject’s written consent to participate in the study, all SAEs, whether related or not related to study drug, are collected, including those thought to be associated with protocol-specified procedures. The investigator should report any SAE occurring after these aforementioned time periods, which is believed to be related to study drug or protocol-specified procedure.
- An SAE report should be completed for any event where doubt exists regarding its seriousness;
- If the investigator believes that an SAE is not related to study drug, but is potentially related to the conditions of the study (such as withdrawal of previous therapy or a complication of a study procedure), the relationship should be specified in the narrative section of the SAE Report Form.For studies with long-term follow-up periods in which safety data are being reported, include the timing of SAE collection.
- The Sponsor will reconcile the clinical database AE cases (**case level only**) transmitted to BMS Global Pharmacovigilance ([Worldwide.Safety@bms.com](mailto:Worldwide.Safety@bms.com)).
  - - The Investigator will request from BMS GPV&E, [aepbusinessprocess@bms.com](mailto:aebusinessprocess@bms.com) the SAE reconciliation report and include the BMS protocol number every 3 months and prior to data base lock or final data summary
    - GPV&E will send the investigator the report to verify and confirm all SAEs have been transmitted to BMS GPV&E.
    - The data elements listed on the GPV&E reconciliation report will be used for case identification purposes. If the Investigator determines a case was not transmitted to BMS GPV&E, the case should be sent immediately to BMS ([Worldwide.Safety@bms.com](mailto:Worldwide.Safety@bms.com)).
- In addition to the Sponsor Investigator’s responsibility to report events to their local HA, suspected serious adverse reactions (whether expected or unexpected) shall be reported by BMS to the relevant competent health authorities in all concerned countries according to local regulations (either as expedited and/or in aggregate reports).
- In accordance with local regulations, BMS will notify sponsor investigators of all reported SAEs that are suspected (related to the investigational product) and unexpected (ie, not previously described in the IB). An event meeting these criteria is termed a Suspected, Unexpected Serious Adverse Reaction (SUSAR). Sponsor investigator notification of these events will be in the form of either a SUSAR Report or a Semi-Annual SUSAR Report.
  - Other important findings which may be reported by BMS as an Expedited Safety Report (ESR) include: increased frequency of a clinically significant expected SAE, an SAE considered associated with study procedures that could modify the conduct of the study, lack of efficacy that poses significant hazard to study subjects, clinically significant safety finding from a nonclinical (eg, animal) study, important safety recommendations from a study data monitoring committee, or sponsor or BMS decision to end or temporarily halt a clinical study for safety reasons.
  - Upon receiving an ESR from BMS, the investigator must review and retain the ESR with the IB. Where required by local regulations or when there is a central IRB/IEC for the study, the sponsor will submit the ESR to the appropriate IRB/IEC. The investigator and IRB/IEC will determine if the informed consent requires revision. The investigator should also comply with the IRB/IEC procedures for reporting any other safety information.

SAEs, whether related or not related to study drug, and pregnancies must be reported to BMS within 24 hours \ 1 Business Day of becoming aware of the event. SAEs must be recorded on either CIOMS, MedWatch, or approved site SAE form.

Pregnancies must be reported and submitted to BMS. BMS will perform due diligence follow-up using the BMS Pregnancy Form which the investigator must complete.

**SAE Email Address:**  Worldwide.Safety@BMS.com

**SAE Facsimile Number:** +1 609-818-3804

If only limited information is initially available, follow-up reports are required. (Note: Follow-up SAE reports should include the same investigator term(s) initially reported.)

If an ongoing SAE changes in its intensity or relationship to study drug or if new information becomes available, a follow-up SAE report should be sent within 24 hours \ 1 Business Day to BMS using the same procedure used for transmitting the initial SAE report.

All SAEs should be followed to resolution or stabilization.

The causal relationship to study drug is determined by a physician and should be used to assess all adverse events (AE). The casual relationship can be one of the following:

Related: There is a reasonable causal relationship between study drug administration and the AE.

Not related: There is not a reasonable causal relationship between study drug administration and the AE.

The term "reasonable causal relationship" means there is evidence to suggest a causal relationship.

Adverse events can be spontaneously reported or elicited during open-ended questioning, examination, or evaluation of a subject. (In order to prevent reporting bias, subjects should not be questioned regarding the specific occurrence of one or more AEs.)

**NONSERIOUS ADVERSE EVENTS**

- Non-serious Adverse Events (AE) are to be provided to BMS in aggregate via interim or final study reports as specified in the agreement or, if a regulatory requirement [eg, IND US trial] as part of an annual reporting requirement.
- Non-serious AE information should also be collected from following the subject’s written consent to participate in the study.

**Non-serious Adverse Event Collection and Reporting**

The collection of non-serious AE information should begin following the subject’s written consent to participate in the study. All non‑serious adverse events (not only those deemed to be treatment-related) should be collected continuously during the treatment period and for a minimum of 100 days following the last dose of study treatment.

Non-serious AEs should be followed to resolution or stabilization, or reported as SAEs if they become serious. Follow-up is also required for non-serious AEs that cause interruption or discontinuation of study drug and for those present at the end of study treatment as appropriate.

Laboratory Test Abnormalities

All laboratory test results captured as part of the study should be recorded following institutional procedures. Test results that constitute SAEs should be documented and reported to BMS as such.

The following laboratory abnormalities should be documented and reported appropriately:

- any laboratory test result that is clinically significant or meets the definition of an SAE
- any laboratory abnormality that required the participant to have study drug discontinued or interrupted
- any laboratory abnormality that required the subject to receive specific corrective therapy.

It is expected that wherever possible, the clinical rather than laboratory term would be used by the reporting investigator (eg, anemia versus low hemoglobin value).

# 10. Central Imaging Review Committee

The central imaging review committee review the imaging of patients to assess response and progression, without assessing to information about treatment.

# 11. Data management and record retention

The study will collect primary data from participating public hospitals or tumor hospitals in China. Electronic case report forms (CRFs) will be used by study investigators or qualified research staff members to enter data. Data will be confidential, in accordance with the applicable regulations. Access to data will be restricted to individuals who are directly involved in the study.

The investigator must retain all study records and source documents for the maximum period required by applicable regulations and guidelines or institution procedures, or for the period specified by the sponsor, whichever is longer.

# 12. Data Monitoring Committee

This study will use an external DMC to perform ongoing safety analyses, an interim efficacy analysis for superiority based on PFS:

• The DMC will review the safety data from approximately the first 20 patients, or approximately 3 months after randomisation of the first patient and then again 6 months later

• The DMC will then meet at least every 6 months to review safety data

• Additional reviews of the safety data may be requested by the DMC at additional points during the study.

This committee will be composed of therapeutic area experts and biostatisticians, who are not employed by BMS and do not have any major conflict of interest.

Following the reviews, the DMC will recommend whether the study should continue unchanged, be stopped, or be modified in any way. Once the DMC has reached a recommendation, a report will be provided to BMS. The report will include the recommendation and any potential protocol amendments, and. The final decision to modify or stop the study will sit with the sponsor. The safety of all BMS clinical studies is closely monitored on an ongoing basis by BMS representatives in consultation with the Patient Safety Department. Issues identified will be addressed; this could involve, for instance, amendments to the clinical study protocol and letters to investigators.

# 13. Statistical Analysis

## 13.1 Sample size

It is estimated that the study would have a power of 90% to detect an extension of the median PFS from 10 months to 18 months, corresponding to a hazard ratio of 0.56, based on a log-rank test with a two-sided significance level of 5%. With 2 years of accrual and 2 years of follow-up time, the number of patients needed for randomization is 150 (75 per group), and the number of PFS events needed for final analysis is 123. With a 10% of dropout rate considered, a total of 168 patients (84 per group) are needed for randomization.

We assume that: 1) About 15% of patients who have received neoadjuvant chemo-NIVO therapy will not receive subsequent CCRT; 2) About 25% of patients who have received CCRT will not be eligible for the randomization. Therefore, 264 patients in total need to be enrolled into neoadjuvant therapy.

We assume one planned interim analysis when approximately 60% of events occur. The threshold of significance, defined by the O’Brien-Fleming type boundary, is 0.0076 in interim analysis and 0.048 for the final analysis.

## 13.2 Statistical plan

### 13.2.1 Description of analysis sets

The Full Analysis Set (FAS) and the Safety Analysis Set described below will be applied to all randomized patients.

Outcome variable: PFS, OS, ORR, populations: ITT

Table: summary of outcome variables and analysis population.

| Outcome variable | Populations |
| --- | --- |
| Efficacy Data |  |
| PFS | ITT |
| OS, ORR, | ITT |
| Safety data |  |
| Adverse events | safety |

1）Full analysis set

Intent-to-treat (ITT): The primary statistical analysis of the efficacy of Nivolumab consolidation versus placebo will include all randomized patients and will compare the treatment arms on the basis of randomized treatment, regardless of the treatment actually receive. Patients who are randomized but do not subsequently go on to receive nivolumab are included in the ITT population.

The statistical analysis of the efficacy of neoadjuvant Nivolumab plus chemotherapy will include all patients enrolled in to the neoadjuvant treatment, regardless of the treatment actually received.

2）Safety analysis set

All patients who received per-protocol treatment and for whom any post-dose data are available will be included in the safety population. Throughout the safety results sections, erroneously treated patients (eg, those randomized to Treatment A but actually given Treatment B) will be accounted for in the actual treatment.

### 13.2.2 Methods of statistical analyses

- 1. PFS

Progression-free survival (PFS) is defined as the time from randomization to the date of the first documented event of tumor progression or death in the absence of disease progression. The interim analysis of the primary endpoint, PFS, will occur when approximately 60% maturity occurs. PFS will be analyzed using a stratified log-rank test adjusting for age at randomization (<65 versus ≥65 years of age), sex (male versus female), smoking history (smoker versus non-smoker) and EGFR mutation status. The effect of treatment will be estimated by the hazard ratio (HR) together with its corresponding 95 % CI and p-value for the ITT population using a stratified Cox regression model.

Subgroup analyses for PFS will be performed (age [<65 years or ≥65 years], sex [male or female], ECOG performance status [0 or 1], smoking history [smoker or non-smoker], disease stage [IIIA, IIIB or IIIC]), histology type [squamous, non-squamous or NOS], EGFR status [mutant or wild type], PD-L1 status [<1%, ≥1% or missing], response to neoadjuvant therapy [PR or SD], response to CCRT [CR, PR or SD]), in which HRs and 95% CIs are calculated using an unstratified Cox regression model.

- 1. OS

OS is defined as the time from randomization until death from any cause. OS will be analyzed using a stratified log-rank test adjusting for age at randomization (<65 versus ≥65 years of age), sex (male versus female), smoking history (smoker versus non-smoker), and EGFR mutation status. The effect of treatment will be estimated by the hazard ratio (HR) together with its corresponding 95 % CI and p-value for the ITT using a stratified Cox regression model.

3）ORR

Tumor response is defined as complete remission (CR), partial remission (PR), stable disease (SD) or progressive disease (PR) according to RECIST version 1.1 (recorded as aggregate response to former therapy), and evaluated after neoadjuvant therapy, 2 months after CCRT and then every 3 months during consolidation or observation, respectively. ORR is defined as the sum of the rates of CR and PR. Response rates are estimated with the use of the Clopper-Pearson method. ORR to neoadjuvant therapy is assessed in patients who have received at least 1 cycle of neoadjuvant therapy. ORR to CCRT is assessed in patients who have undergone CCRT. Overall ORR with consolidative nivolumab or observation is assessed in patients who are randomized. Patients with missing ORR data are counted as non-responders.

1. Safety data

AEs will be listed individually by patient. The number of patients experiencing each AE will be summarized by treatment arm and CTCAE grade. Toxicities during the neoadjuvant period include those occurring from the first neoadjuvant dose to the initiation of CCRT. Toxicities during the CCRT period include those occurring from the initiation of CCRT to the time of randomization. Post-randomization toxicities in the consolidation group include those occurring from the time of randomization to 3 months after the last dose of consolidative nivolumab, or the initiation of a new anti-cancer therapy, whichever comes first. Post-randomization toxicities in the observation group include those occurring from the time of randomization to 15 months thereafter, or the initiation of a new anti-cancer therapy, whichever comes first.

# Reference List

1 Torre, L. A. *et al.* Global cancer statistics, 2012. *CA Cancer J Clin* 65, 87-108 (2015).

2 Goldstraw, P. *et al.* The IASLC Lung Cancer Staging Project: Proposals for Revision of the TNM Stage Groupings in the Forthcoming (Eighth) Edition of the TNM Classification for Lung Cancer. *J Thorac Oncol* 11, 39-51 (2016).

3 Dillman, R. O. *et al.* A randomized trial of induction chemotherapy plus high-dose radiation versus radiation alone in stage III non-small-cell lung cancer. *N Engl J Med* 323, 940-945 (1990).

4 Sause, W. *et al.* Final results of phase III trial in regionally advanced unresectable non-small cell lung cancer: Radiation Therapy Oncology Group, Eastern Cooperative Oncology Group, and Southwest Oncology Group. *Chest* 117, 358-364 (2000).

5 Le Chevalier, T. *et al.* Radiotherapy alone versus combined chemotherapy and radiotherapy in nonresectable non-small-cell lung cancer: first analysis of a randomized trial in 353 patients. *J Natl Cancer Inst* 83, 417-423 (1991).

6 Schaake-Koning, C. *et al.* Effects of concomitant cisplatin and radiotherapy on inoperable non-small-cell lung cancer. *N Engl J Med* 326, 524-530 (1992).

7 Jeremic, B., Shibamoto, Y., Acimovic, L. & Djuric, L. Randomized trial of hyperfractionated radiation therapy with or without concurrent chemotherapy for stage III non-small-cell lung cancer. *J Clin Oncol* 13, 452-458 (1995).

8 Curran, W. J., Jr. *et al.* Sequential vs. concurrent chemoradiation for stage III non-small cell lung cancer: randomized phase III trial RTOG 9410. *J Natl Cancer Inst* 103, 1452-1460 (2011).

9 Furuse, K. *et al.* Phase III study of concurrent versus sequential thoracic radiotherapy in combination with mitomycin, vindesine, and cisplatin in unresectable stage III non-small-cell lung cancer. *J Clin Oncol* 17, 2692-2699 (1999).

10 Aupérin, A. *et al.* Concomitant radio-chemotherapy based on platin compounds in patients with locally advanced non-small cell lung cancer (NSCLC): a meta-analysis of individual data from 1764 patients. *Ann Oncol* 17, 473-483 (2006).

11 Wang, Y. *et al.* Combining Immunotherapy and Radiotherapy for Cancer Treatment: Current Challenges and Future Directions. *Front Pharmacol* 9, 185 (2018).

12 Sacco, P. C., Maione, P., Guida, C. & Gridelli, C. The Combination of New Immunotherapy and Radiotherapy: A N ew Potential Treatment for Locally Advanced Non-Small Cell Lung Cancer. *Curr Clin Pharmacol* 12, 4-10 (2017).

13 Antonia, S. J. *et al.* Durvalumab after Chemoradiotherapy in Stage III Non-Small-Cell Lung Cancer. *N Engl J Med* 377, 1919-1929 (2017).

14 Antonia, S. J. *et al.* Overall Survival with Durvalumab after Chemoradiotherapy in Stage III NSCLC. *N Engl J Med* 379, 2342-2350 (2018).

15 Forde, P. M. *et al.* Neoadjuvant PD-1 Blockade in Resectable Lung Cancer. *N Engl J Med* 378, 1976-1986 (2018).

16 Werner-Wasik, M. *et al.* Increasing tumor volume is predictive of poor overall and progression-free survival: secondary analysis of the Radiation Therapy Oncology Group 93-11 phase I-II radiation dose-escalation study in patients with inoperable non-small-cell lung cancer. *Int J Radiat Oncol Biol Phys* 70, 385-390 (2008).

17 Basaki, K. *et al.* Prognostic factors for survival in stage III non-small-cell lung cancer treated with definitive radiation therapy: impact of tumor volume. *Int J Radiat Oncol Biol Phys* 64, 449-454 (2006).

18 Raben, D. *et al.* Patterns of Disease Progression with Durvalumab in Stage III Non-small Cell Lung Cancer (PACIFIC). *International Journal of Radiation Oncology, Biology, Physics* 105, 683 (2019).

19 Kong, F. M. *et al.* Effect of Midtreatment PET/CT-Adapted Radiation Therapy With Concurrent Chemotherapy in Patients With Locally Advanced Non-Small-Cell Lung Cancer: A Phase 2 Clinical Trial. *JAMA Oncol* 3, 1358-1365 (2017).

20 Wild, A. T. *et al.* Lymphocyte-Sparing Effect of Stereotactic Body Radiation Therapy in Patients With Unresectable Pancreatic Cancer. *Int J Radiat Oncol Biol Phys* 94, 571-579 (2016).

21 Tang, C. *et al.* Lymphopenia association with gross tumor volume and lung V5 and its effects on non-small cell lung cancer patient outcomes. *Int J Radiat Oncol Biol Phys* 89, 1084-1091 (2014).
