# Supplementary material for: Consolidative nivolumab versus observation in unresectable stage III non-small cell lung cancer patients following neoadjuvant nivolumab plus chemotherapy and concurrent chemoradiotherapy (CA209-7AL): a randomized clinical trial
Source: Signal Transduct Target Ther. 2025 Sep 29;10:317. doi: 10.1038/s41392-025-02408-3 (PMC12477293; doi:10.1038/s41392-025-02408-3)
Supplement: Supplementary file 2 — Supplement 2. Supplementary Tables and figures [file 41392_2025_2408_MOESM2_ESM.docx]

Supplementary Materials for

**Consolidative Nivolumab versus observation in Unresectable Stage III Non-Small Cell Lung Cancer Post Neoadjuvant Nivolumab plus Chemotherapy and Concurrent Chemoradiotherapy (CA209-7AL): A Randomized Clinical Trial**

Bo Qiu, YuanYuan Zhao, WenZhuo He, WeiJin Zeng, HongMei Zhang, WeiNeng Feng, Jun Jia, DaoDu Wang, DaQuan Wang, FangJie Liu, SongRan Liu, ShaoHan Yin, ChuanMiao Xie, Rui Zhou, Yi Hu, QianWen Liu, JinYu Guo, SuPing Guo, YingJia Wu, QiaoTing Luo, JiBin Li, YunPeng Yang, LiangPing Xia, Li Zhang, Hui Liu

#Correspondence to:

Prof Hui Liu, [liuhui@sysucc.org.cn](mailto:liuhui@sysucc.org.cn)

Prof Li Zhang, [zhangli@sysucc.org.cn](mailto:zhangli@sysucc.org.cn)

Prof LiangPing Xia, xialp@sysucc.org.cn

**This PDF file includes:**

Figures. S1 to S9

Tables. S1 to S11

**
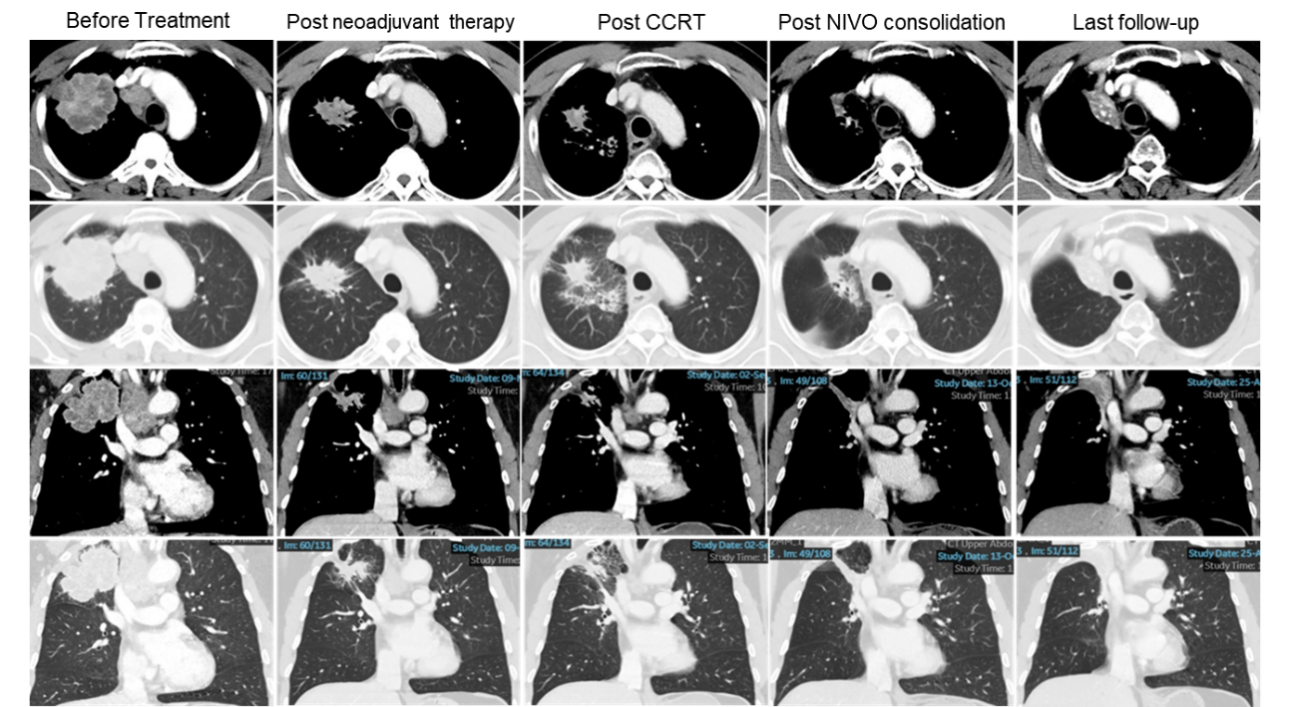
a.**

**b.**


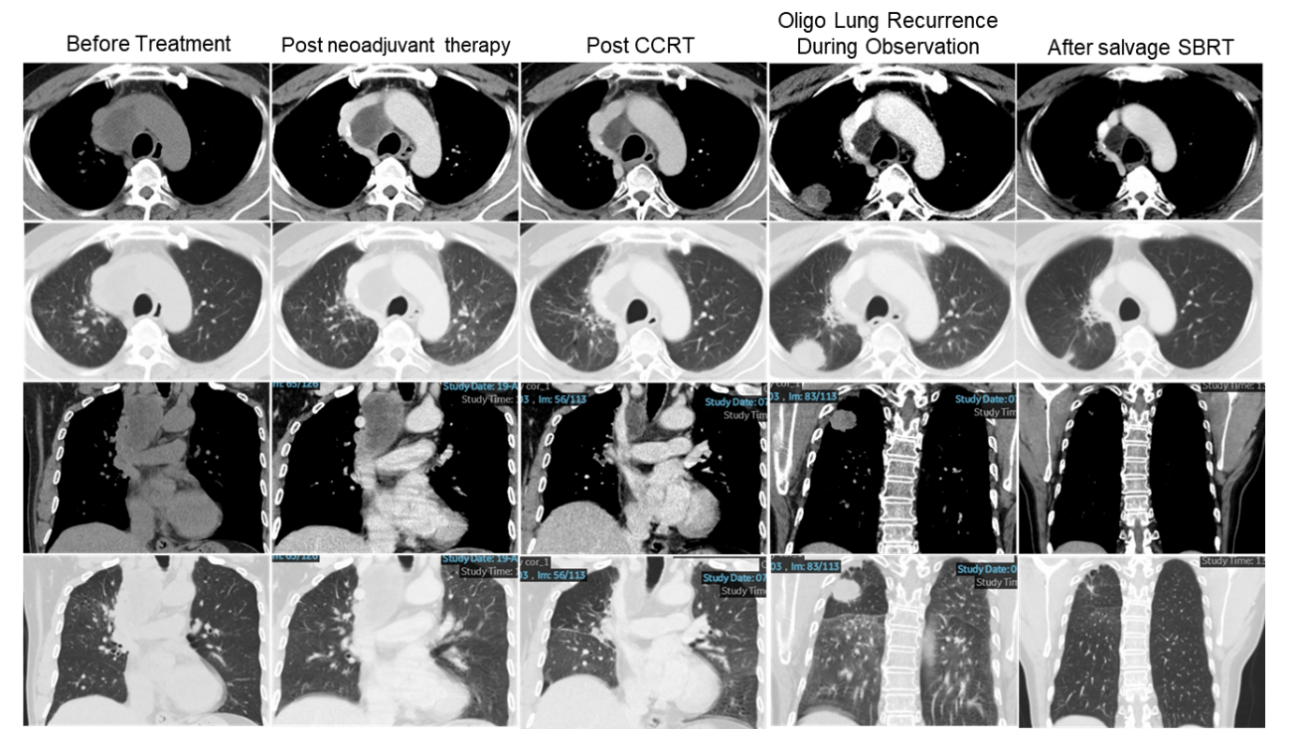


**Figure S1. The CT imaging of two typical patients along the treatment course in CA209-7AL. a:** the patient with cT4N3M0 stage IIIC disease in consolidation group, who had partial remission after neoadjuvant therapy and CCRT and achieved complete remission following nivolumab consolidation. **b**: the patient with cT1N3M0 stage IIIB disease in observation group, who had stable disease after neoadjuvant therapy and partial remission after CCRT. Local recurrence occurred during the observation period, the patient had salvage SBRT and remained disease free at the date of data cut-off.

**a**

**
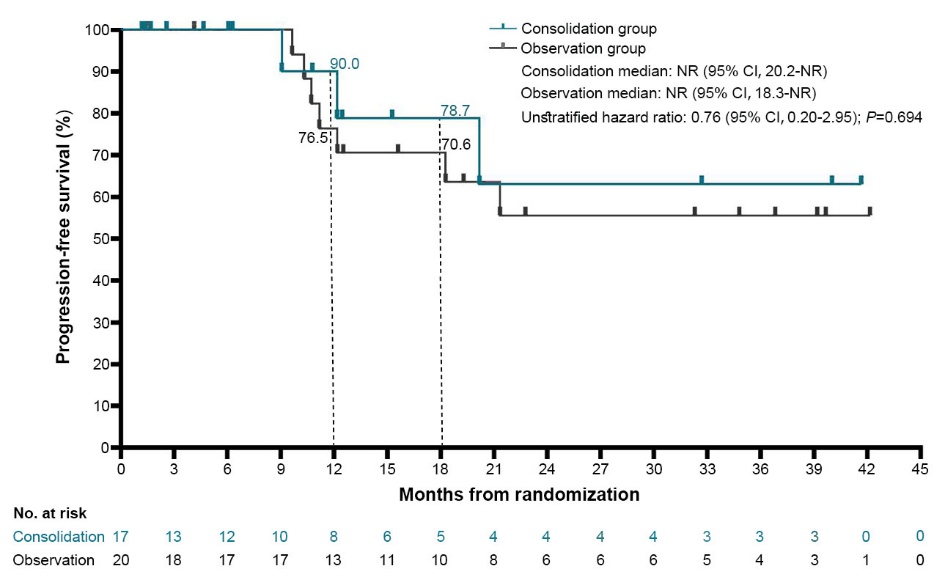
**

**b**

**
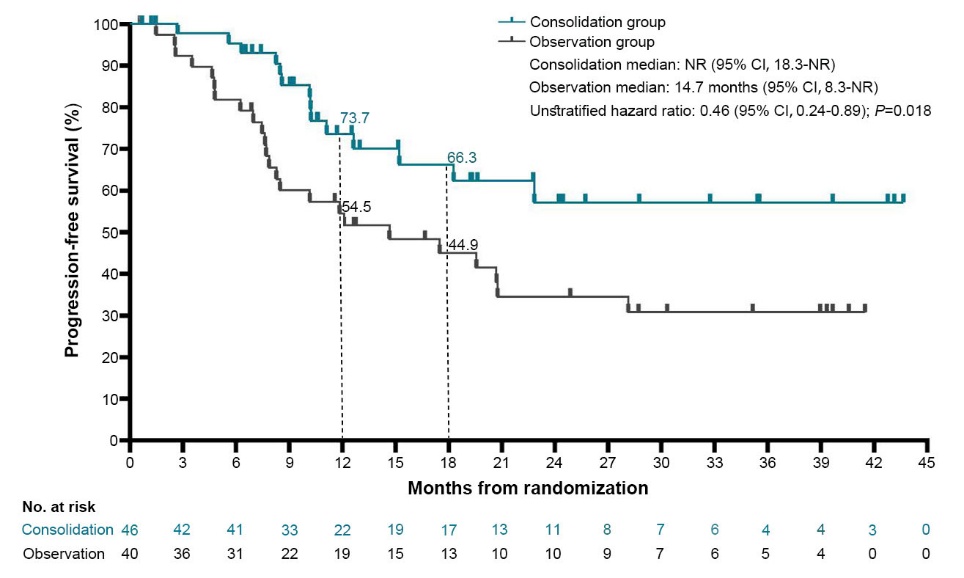
**

**c**

**
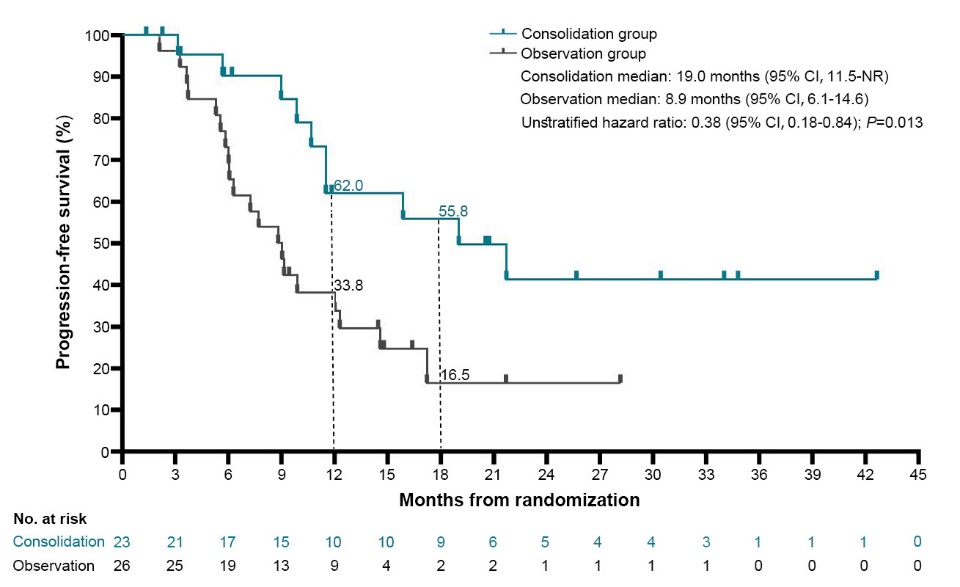
**

**Figure S2. Progression-free survival by baseline stage of disease in the randomized population of CA209-7AL.**

Panel a depicts PFS in patients with baseline disease stage IIIA. Panel b depicts PFS in patients with baseline disease stage IIIB. Panel C depicts PFS in patients with baseline disease stage IIIC.

**a**


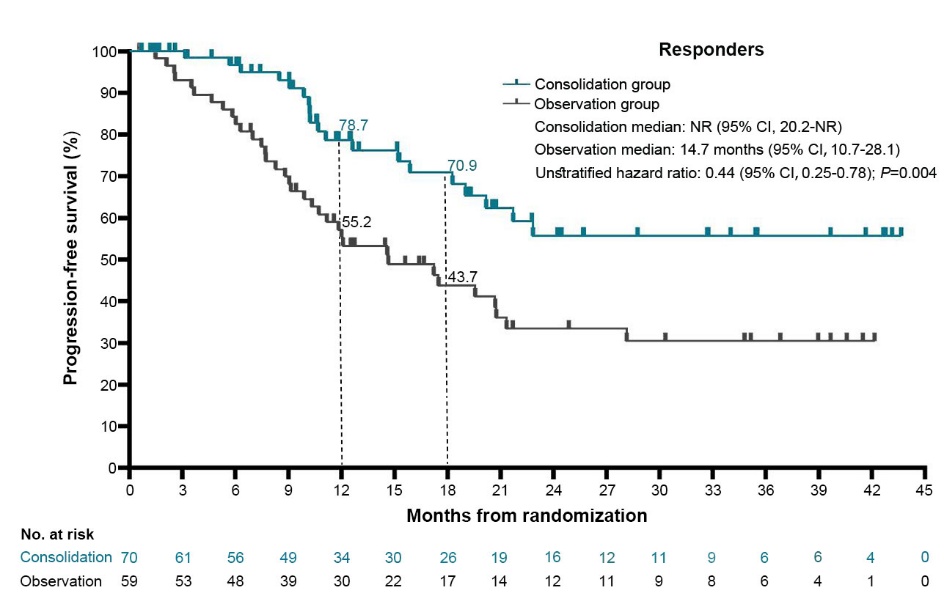


**b**


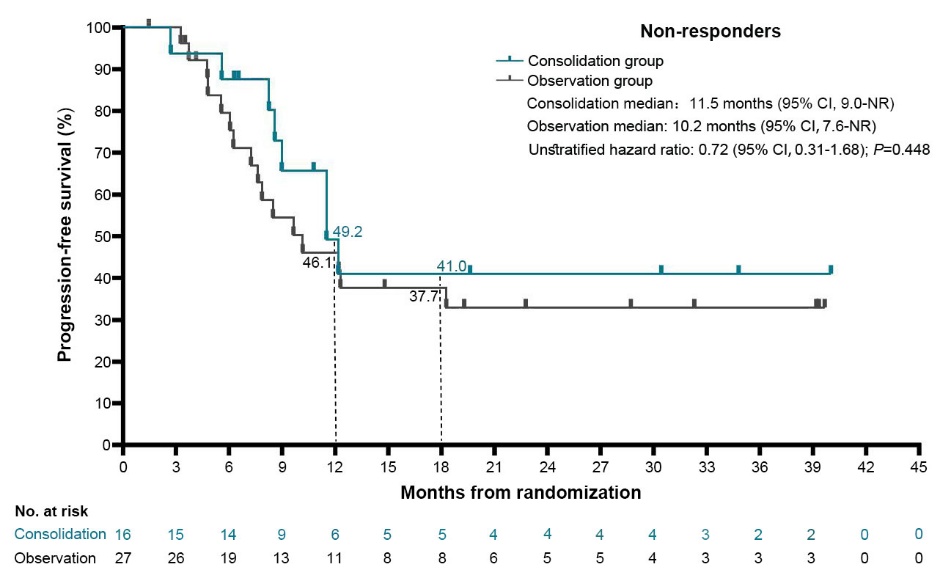


**Figure S3. Progression-free survival by responses to prior neoadjuvant therapy in the randomized population of CA209-7AL.** Panel a depicts PFS in patients with partial remission to neoadjuvant therapy (responders). Panel b depicts PFS in patients with stable disease to neoadjuvant therapy (non-responders).

**
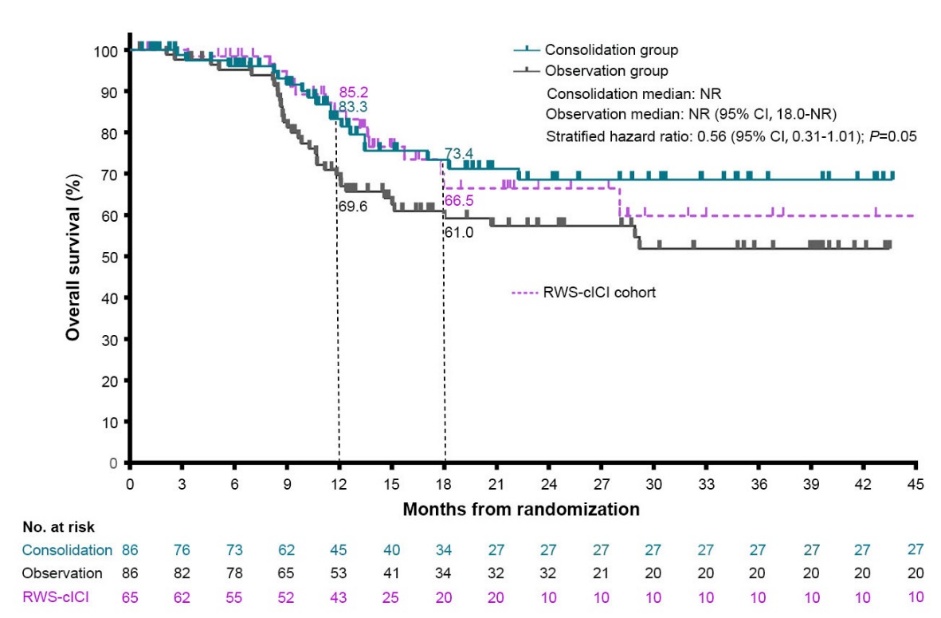
**

**Figure S4. Overall survival in the randomized population of CA209-7AL and the RWS-cICI cohort.**

RWS-cICI cohort: patients who received consolidative immunotherapy in the real-world study.

**
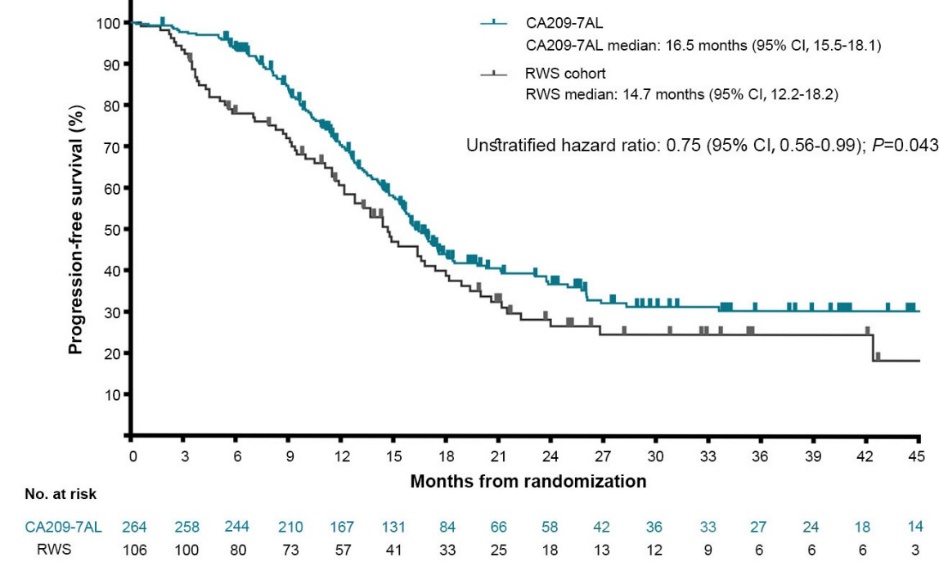
**

**Figure S5. The real-world progression-free survival in all patients from CA209-7AL and the RWS cohort. Real-world PFS was measured from the time of screening.**

**
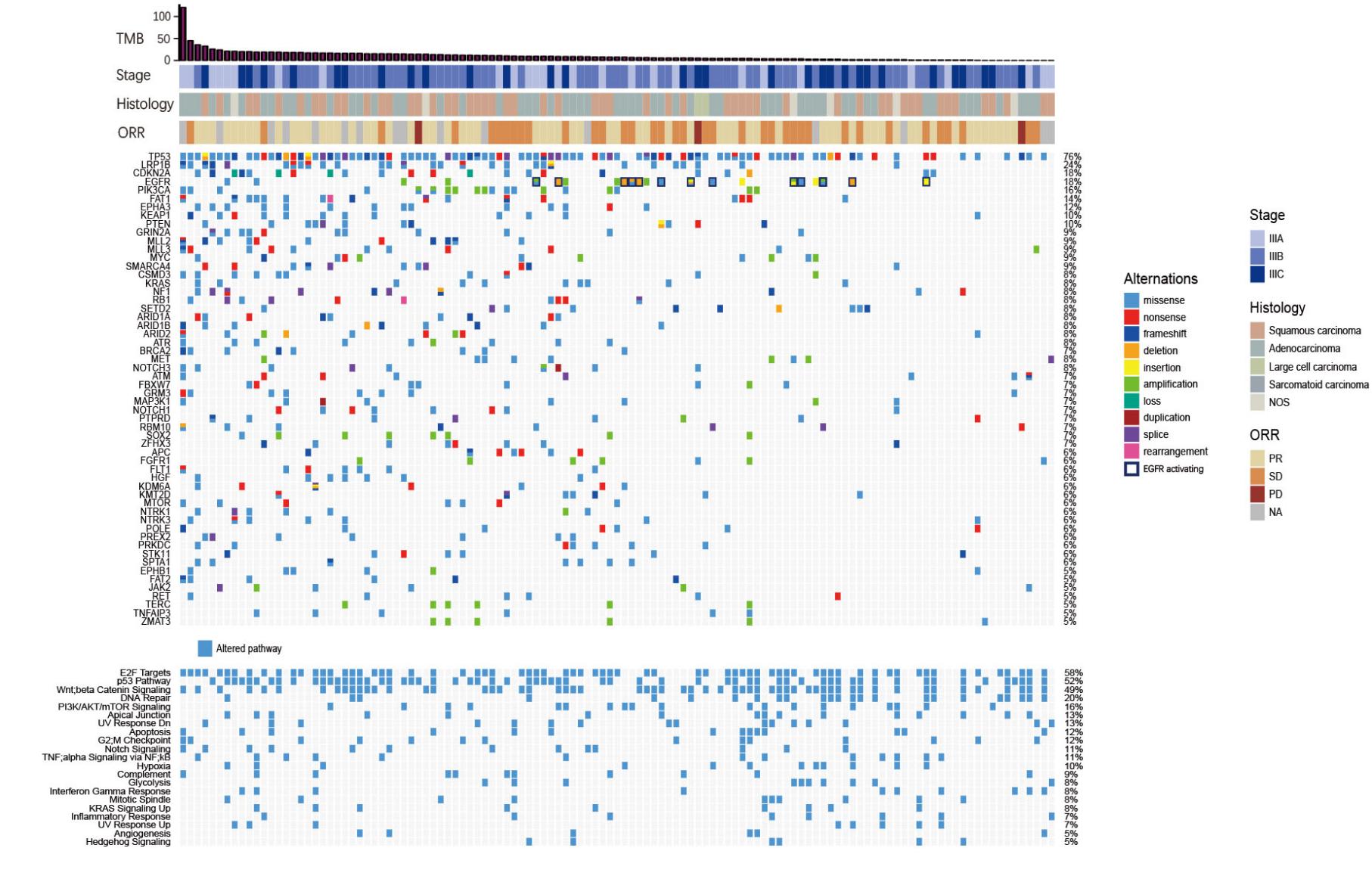
Figure S6. Gene sequencing results with qualified samples from 106 patients in CA209-7AL and 13 patients in the RWS cohort.** ORR, objective response rate; NOS, not other specified; PR, partial remission; SD, stable disease; PD, progressive disease; NA, not applicable; TMB, tumor mutational burden.

**
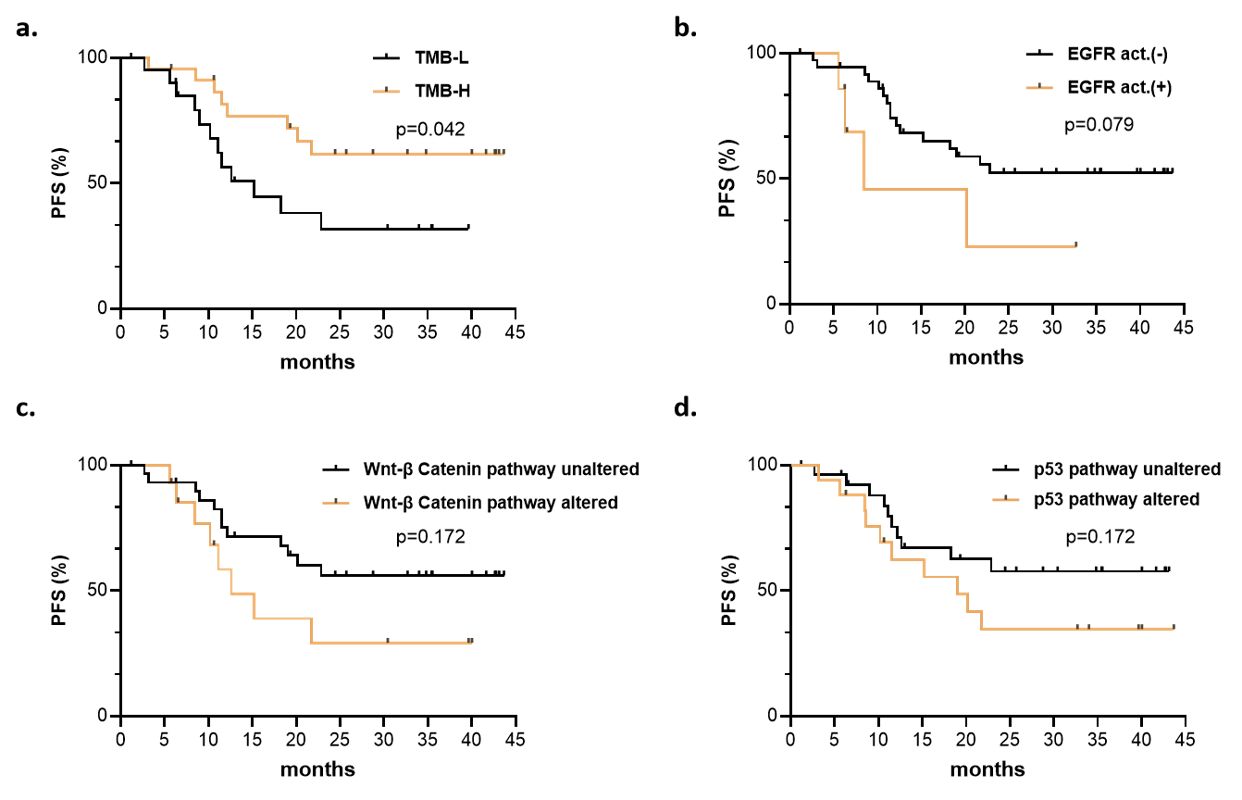
**

**Figure S7. The association of TMB, individual somatic mutations, and pathways with treatment outcomes in CA209-7AL.** In the consolidation group, patients with TMB-H demonstrated longer progression-free survival (PFS) compared to those with low tumor mutational burden (TMB-L) (a); PFS was reduced in patients with *EGFR* activating mutations (b), alterations in the Wnt-β Catenin signaling pathway (c), or alterations in the p53 pathway (d), with marginal statistical significance observed.

**
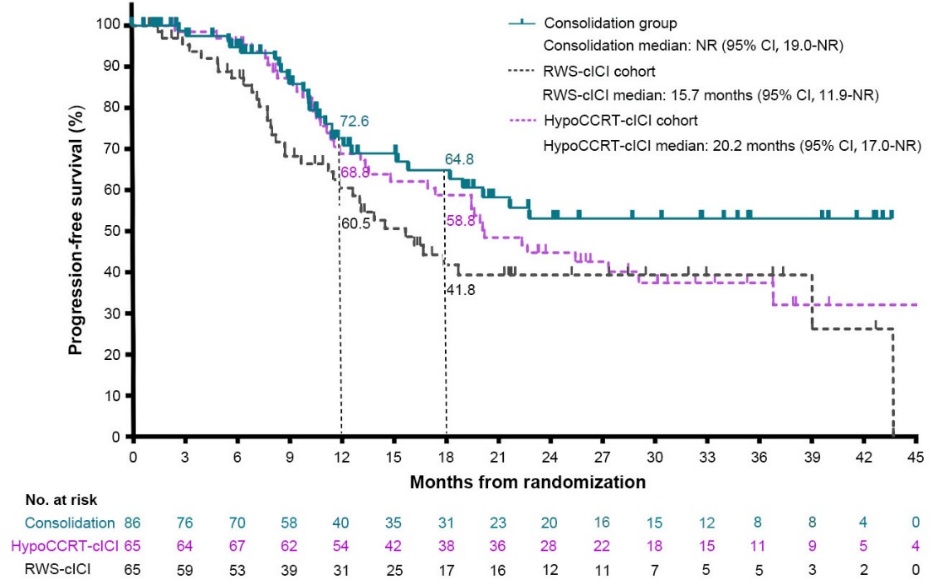
**

**Figure S8. Kaplan-Meier curves for progression-free survival** **in the NIVO consolidation group of CA209-7AL (N=86), the RWS-cICI cohort (N=65), and the hypoCCRT-cICI cohort (N=65).** cICI, consolidative immunotherapy; hypoCCRT, hypofractionated radiotherapy with concurrent chemotherapy; RWS, real-word study.


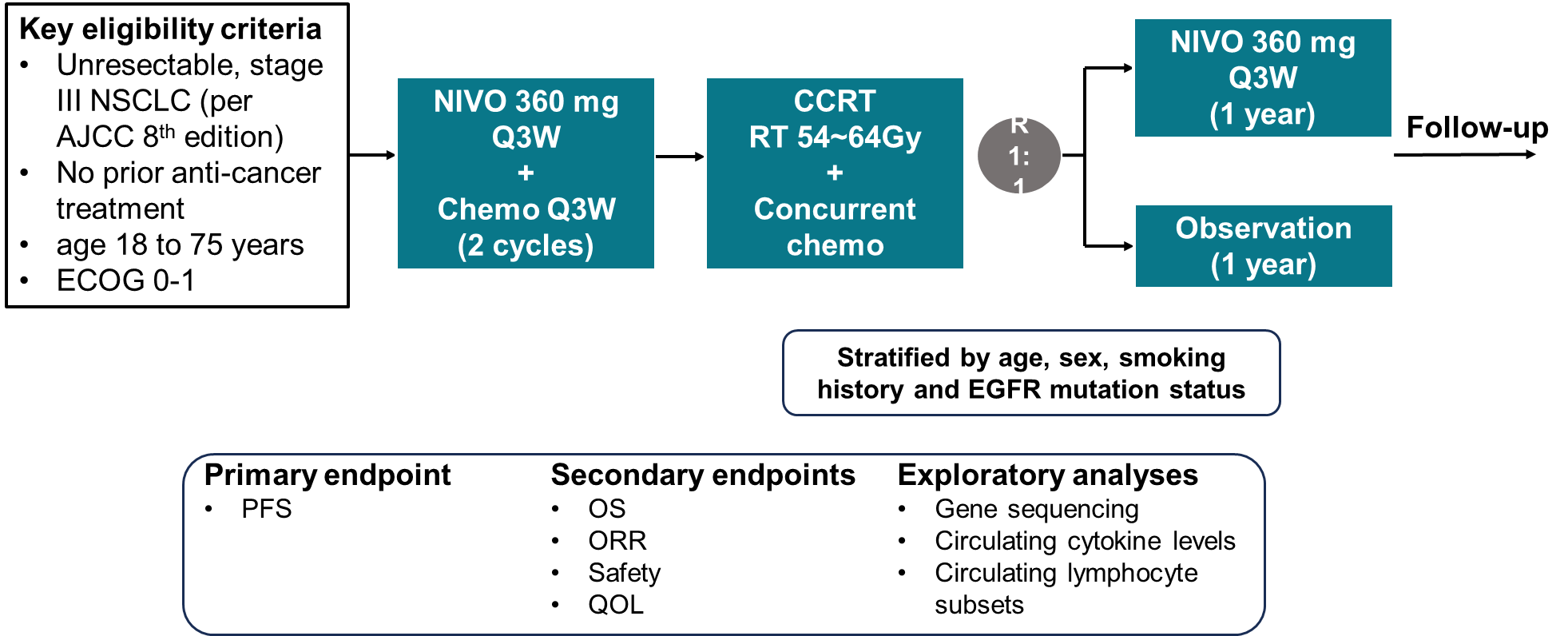


**Figure S9. Study design of the phase II, randomized trial CA209-7AL.** NSCLC, non-small cell lung cancer; ECOG PS, Eastern Cooperative Oncology Group performance status; NIVO, nivolumab; CCRT, concurrent chemoradiotherapy; PFS, progression-free survival; OS, overall survival; ORR, objective response rate; QOL, quality of life.

**Table S1. Radiation treatment plan and dose distribution of radiotherapy in CA209-7AL (N=242).**

| Treatment plans and dose distributions of CCRT | No. (%) |
| --- | --- |
| Prescription dose and fractions | ----- |
| 40Gy/10fr + 24Gy/6fr | 81 (33.5%) |
| 30Gy/6fr + 24~30Gy/6fr | 161 (66.5%) |
| GTV volume, cc, median (IQR) | 21.4 (10.8~36.6) |
| V_prescription dose_ of PTV_GTV, %, median (IQR) | 87.6 (81.0~93.31) |
| V20 of lungs, %, median (IQR) | 23.5 (19.5~26.6) |
| Mean lung dose, EQD2, Gy, median (IQR) | 20.5 (17.6~23.6) |
| Mean heart dose, EQD2, Gy, median (IQR) | 10.6 (6.3~18.9) |
| Maximum esophagus dose, EQD2, Gy, median (IQR) | 69.4 (67.1~75.1) |
| Maximum spinal cord dose, EQD2, Gy, median (IQR) | 35.3 (32.8~39.2) |
| Maximum dose of proximal bronchial tree, EQD2, Gy, median (IQR) | 86.8 (82.5~91.8) |

EQD2, Equivalent dose in 2 Gy/f; GTV, gross tumor volume; IQR, interquartile range; PTV, planning target volume; VXX, the percentage volume irradiated above XX Gy.

**Table S2. Summary of progression pattern in randomized population of CA209-7AL and the RWS-cICI cohort.**

|  | CA209-7AL | | | RWS |
| --- | --- | --- | --- | --- |
|  | Randomized population  (N=172) | Consolidation group  (N=86) | Observation group  (N=86) | RWS-cICI cohort  (N=65) |
| Disease progression | 56 (32.6%) | 15 (17.4%) | 41 (47.7%) | 28 (43.1%) |
| Locoregional | 32 (18.6%) | 11 (12.8%) | 21 (24.4%) | 21 (32.3%) |
| CCRT In-field | 25 (14.5%) | 7 (8.1%) | 18 (20.9%) | 19 (29.2%) |
| CCRT Out-field | 5 (2.9%) | 3 (3.5%) | 2 (2.3%) | 1 (1.5%) |
| Simultaneous in-/out-field | 2 (1.2%) | 1 (1.2%) | 1 (1.2%) | 1 (1.5%) |
| Distant | 32 (18.6%) | 8 (9.3%) | 24 (27.9%) | 9 (13.8%) |
| Oligo | 23 (13.4%) | 6 (7.0%) | 17 (19.8%) | 5 (7.7%) |
| Multiple | 9 (5.2%) | 2 (2.3%) | 7 (8.1%) | 4 (6.2%) |

CCRT, concurrent chemoradiotherapy; RWS-cICI cohort: patients who received consolidative immunotherapy in the real-world study.

**Table S3. Summary of post-progression salvage treatment in progressed patients in randomized population of CA209-7AL and the RWS-cICI cohort.**

|  | CA209-7AL | | | RWS |
| --- | --- | --- | --- | --- |
|  | Randomized population  (N=56) | Consolidation group  (N=15) | Observation group  (N=41) | RWS-cICI cohort  (N=28) |
| Received salvage treatment | 45 (80.4%) | 12 (80.0%) | 33 (80.5%) | 22 (78.6%) |
| Chemotherapy | 12 (21.4%) | 1 (6.7%) | 11 (26.8%) | 2 (7.1%) |
| Chemo-immunotherapy | 12 (21.4%) | 6 (40.0%) | 6 (14.6%) | 9 (32.1%) |
| TKI treatment | 8 (14.3%) | 3 (20.0%) | 5 (12.2%) | 2 (7.1%) |
| Immunotherapy | 2 (3.6%) | 1 (6.7%) | 1 (2.4%) | 4 (14.3%) |
| Radiotherapy | 9 (16.1%) | 1 (6.7%) | 8 (19.5%) | 5 (17.9%) |
| Concurrent Chemoradiotherapy | 1 (1.8%) | 0 | 1 (2.4%) | 0 |
| Surgery | 1 (1.8%) | 0 | 1 (2.4%) | 0 |

TKI, tyrosine kinase inhibitor; RWS-cICI cohort: patients who received consolidative immunotherapy in the real-world study.

**Table S4. Adverse events during neoadjuvant period (N=264) and CCRT period (N=242) in CA209-7AL.**

| Adverse Event during neoadjuvant period (N=264) | No. (%) | |
| --- | --- | --- |
| Led to discontinuation of neoadjuvant therapy | 19 (7.2%) | |
|  | Any Grade | Grade 3 or 4 |
| All | 259 (98.1%) | 120 (45.5%) |
| Lymphopenia | 214 (81.1%) | 93 (35.2%) |
| Neutrophil count decreased | 35 (13.3%) | 26 (9.9%) |
| Leukopenia | 41 (15.5%) | 19 (7.2%) |
| Platelet count decreased | 37 (14.0%) | 13 (4.9%) |
| Diarrhea | 55 (20.8%) | 11 (4.2%) |
| Hyponatremia | 116 (43.9%) | 10 (3.8%) |
| Pneumonitis | 11 (4.2%) | 3 (1.1%) |
| Vomiting | 23 (8.7%) | 3 (1.1%) |
| Aspartate aminotransferase increased | 31 (11.7%) | 3 (1.1%) |
| Alanine aminotransferase increased | 57 (21.6%) | 2 (0.8%) |
| Dyspnea | 19 (7.2%) | 2 (0.8%) |
| Fever | 21 (8.0%) | 2 (0.8%) |
| Hemoglobin decreased | 170 (64.4%) | 1 (0.4%) |
| Anorexia | 75 (28.4%) | 1 (0.4%) |
| Nausea | 19 (7.2%) | 1 (0.4%) |
| Rash | 17 (6.4%) | 1 (0.4%) |
| Fatigue | 17 (6.4%) | 1 (0.4%) |
| **Adverse Event during CCRT period (N=242)** | No. (%) | |
| Led to discontinuation of CCRT | 5 (2.1%) | |
|  | Any Grade | Grade 3 or 4 |
| All | 238 (98.3%) | 99 (40.9%) |
| Lymphopenia | 218 (90.1%) | 89 (36.8%) |
| Esophagitis | 42 (17.4%) | 8 (3.3%) |
| Pneumonitis | 132 (54.5%) | 6 (2.5%) |
| Dyspnea | 38 (15.7%) | 5 (2.1%) |
| Aspartate aminotransferase increased | 29 (12.0%) | 5 (2.1%) |
| Alanine aminotransferase increased | 24 (9.9%) | 4 (1.7%) |
| Leukopenia | 88 (36.4%) | 4 (1.7%) |
| Cough | 74 (30.6%) | 3 (1.2%) |
| Neutrophil count decreased | 24 (22.3%) | 3 (1.2%) |
| Hemoglobin decreased | 100 (41.3%) | 2 (0.8%) |
| Anorexia | 45 (18.6%) | 0 |

Included were events reported in at least 10% of the patients and all grade 3~4 events. No Grade 5 adverse event was recorded. CCRT, concurrent chemoradiotherapy.

**Table S5. Immune-related toxicities in the consolidation group (N=86).**

| Immune-related toxicities | No. (N/86) | |
| --- | --- | --- |
| Led to discontinuation of consolidation | 13 (15.1%) | |
|  | Any grade | Grade 3-5 |
| All | 27 (31.4%) | 2 (2.3%) |
| Pneumonitis | 14 (16.3%) | 2 (2.3%) |
| Alanine aminotransferase increased | 10 (11.6%) | 0 |
| Aspartate aminotransferase increased | 7 (8.1%) | 0 |
| Rash | 6 (7.0%) | 0 |
| Hypothyroidism | 5 (5.8%) | 0 |
| Musculoskeletal pain | 2 (2.3%) | 0 |
| Colitis | 1 (1.2%) | 0 |

**Table S6. Treatment details in the RWS cohort (N=106).**

| Treatment details | No. (%) |
| --- | --- |
| Radiation dose, Gy, median (IQR) | 63 (60-64) |
| Concurrent chemotherapy |  |
| Taxol+platinum | 79 (74.5%) |
| Pemetrexed+platinum | 27 (25.5%) |
| Consolidative immunotherapy | 65 (61.3%) |
| Durvalumab | 26 (24.5%) |
| Tislelizumab | 19 (17.9%) |
| Sintilimab | 11 (10.4%) |
| Others | 9 (8.5%) |

IQR, interquartile range; RWS, real-word study.

**Table S7. Adverse events during CCRT (N=106) and cICI period (N=65) in the RWS cohort.**

| Adverse Event during CCRT (N=106) | No. (%) | |
| --- | --- | --- |
| Led to discontinuation of CCRT | 4 (3.8%) | |
|  | Any Grade | Grade 3 or 4 |
| All | 104 (98.1%) | 57 (53.8%) |
| Lymphopenia | 104 (98.1%) | 55 (52.9%) |
| Esophagitis | 37 (34.9%) | 12 (11.3%) |
| Pneumonitis | 74 (69.8%) | 4 (3.8%) |
| Cough | 55 (51.9%) | 4 (3.8%) |
| Dyspnea | 20 (18.9%) | 3 (2.8%) |
| Aspartate aminotransferase increased | 12 (11.3%) | 3 (2.8%) |
| Thrombocytopenia | 12 (11.3%) | 5 (4.7%) |
| Neutrophil count decreased | 35 (33.0%) | 3 (2.8%) |
| Hemoglobin decreased | 57 (53.8%) | 2 (1.9%) |
| Dermatitis | 6 (5.7%) | 1 (0.9%) |
| Anorexia | 22 (20.8%) | 0 |
| **Adverse Event during cICI (N=65)** | No. (%) | |
| Led to discontinuation of cICI | 22 (33.8%) | |
|  | Any Grade | Grade 3 |
| All | 50 (76.9%) | 10 (15.4%) |
| Dyspnea | 11 (16.9%) | 3 (4.6%) |
| Pneumonitis | 25 (38.5%) | 2 (3.1%) |
| Lymphopenia | 13 (20.0%) | 3 (4.6%) |
| Cough | 26 (30.2%) | 2 (3.1%) |
| Proximal bronchial  tree toxicity | 12 (18.5%) | 1 (1.5%) |
| Thrombocytopenia | 3 (4.6%) | 1 (1.5%) |
| Diarrhea | 3 (4.6%) | 1 (1.5%) |
| Hemoglobin  decreased | 9 (10.5%) | 1 (1.5%) |
| Alanine  aminotransferase  increased | 7 (9.2%) | 0 |
| Aspartate  aminotransferase  increased | 6 (8.1%) | 0 |
| Hypothyroidism | 5 (5.8%) | 0 |

Included events during CCRT were events reported in at least 10% of the patients and all grade 3~4 events. Included events during cICI were events reported in at least 5% of the patients and all grade 3~4 events. No Grade 5 adverse event was recorded. CCRT, concurrent chemoradiotherapy; RWS, real-world study.

**Table S8. Radiation dose constraints for organs at risk in CA209-7AL.**

| Organs at risk | Dose constraints | | | |
| --- | --- | --- | --- | --- |
|  | Dose regimen 1 | | Dose regimen 2 | |
|  | The first course  (40Gy/10Fr) | The boost course  (24Gy/6Fr) | The first course  (30Gy/6Fr) | The boost course  (24~30Gy/6Fr) |
| Lungs | V15≤20% | V10≤10% | V15≤10% | V15≤10% |
| Ipsilateral lung | V15≤25% | V10≤15% | V15≤20% | V15≤20% |
| Spinal cord | Dmax≤20Gy | Dmax≤10Gy | Dmax≤12Gy | Dmax≤12Gy |
| Heart | V20≤20% | V10≤10% | V20≤15% | V20≤15% |
| Esophagus | Dmax≤36Gy,  V30≤20% | Dmax≤20Gy,  V10≤20% | Dmax≤25Gy,  V20≤20% | Dmax≤24Gy,  V20≤20% |
| Great vessels | Dmax≤44Gy,  V40≤10cc | Dmax≤22Gy,  V20≤10cc | Dmax≤33Gy,  V30≤10cc | Dmax≤33Gy,  V30≤10cc |
| Proximal bronchial tree | Dmax≤44Gy | Dmax≤22Gy | Dmax≤33Gy | Dmax≤26.4Gy |

Fr, fraction; VXX, the percentage volume irradiated above XX Gy; Dmax, maximum dose.

**Table S9.** **Baseline characteristics of patients** **in** **the NIVO consolidation group of CA209-7AL (N=86), the RWS-cICI cohort (N=65), and the** **hypoCCRT-cICI cohort (N=65).**

|  | NIVO Consolidation group (N=86) | RWS-cICI cohort (N=65) | HypoCCRT-cICI cohort (N=65) |
| --- | --- | --- | --- |
| Age, years | 57 (39-73) | 58 (39-75) | 56 (37-75) |
| < 65 | 56 (65.1%) | 47 (72.3%) | 47 (72.3%) |
| ≥65 | 30 (34.9) | 18 (27.7%) | 18 (27.7%) |
| Sex |  |  |  |
| Male | 71 (82.6%) | 54 (83.1%) | 55 (84.6%) |
| Female | 15 (17.4%) | 11 (16.9%) | 10 (15.4%) |
| ECOG PS |  |  |  |
| 0 | 57 (66.3%) | 40 (61.5%) | 42 (64.6%) |
| 1 | 29 (33.7%) | 25 (38.5%) | 23 (35.4%) |
| Smoking history |  |  |  |
| Non-smoker | 28 (32.6%) | 23 (35.4%) | 23 (35.4%) |
| Smoker | 58 (67.4%) | 42 (64.6%) | 42 (64.6%) |
| UICC/AJCC Stage (8th) |  |  |  |
| IIIA | 17 (19.8%) | 16 (24.6%) | 15 (23.1%) |
| IIIB | 46 (53.5%) | 30 (46.2%) | 33 (50.8%) |
| IIIC | 23 (26.7%) | 19 (29.2%) | 17 (26.1%) |
| Histology |  |  |  |
| Squamous | 38 (44.2%) | 37 (56.9%) | 34 (52.3%) |
| Non-squamous | 43 (50.0%) | 26 (40.0%) | 27 (41.5%) |
| NOS | 5 (5.8%) | 2 (3.1%) | 4 (6.2%) |
| EGFR status |  |  |  |
| Wild | 78 (90.7%) | 61 (93.8%) | 60 (92.3%) |
| Mutant | 8 (9.3%) | 4 (6.2%) | 5 (7.7%) |
| PD-L1 expression† |  |  |  |
| < 1% | 23 (26.7%) | 14 (21.5%) | 18 (27.7%) |
| ≥1% | 38 (44.2%) | 26 (40.0%) | 24 (36.9%) |
| Missing | 25 (29.1%) | 25 (38.5%) | 23 (35.4%) |

Data are median (range) or n (%). cICI, consolidative immunotherapy; ECOG PS, Eastern Cooperative Oncology Group performance status; EGFR, epidermal growth factor receptor; hypo-CCRT, hypofractionated radiotherapy with concurrent chemotherapy; NOS, Not Otherwise Specified; † Determined by the PD-L1 IHC C22C3 pharmDx assay. Assessment of baseline PD-L1 expression was not mandatory for study enrolment. RWS, real-world study.

**Table S10. Treatment details** **in the hypoCCRT-cICI cohort (N=65).**

| Treatment details | No. (%) |
| --- | --- |
| Prescription dose and fractions |  |
| 40Gy/10fr + 24Gy/6fr | 17 (26.2%) |
| 30Gy/6fr + 24~30Gy/6fr | 48 (73.8%) |
| Concurrent chemotherapy |  |
| Docetaxel+platinum | 21 (32.3%) |
| Nab-paclitaxel+platinum | 44 (67.7%) |
| cICI |  |
| Durvalumab | 20 (30.8%) |
| Tislelizumab | 30 (46.1%) |
| Sintilimab | 8 (12.3%) |
| Others | 7 (10.8%) |
| The status of cICI |  |
| Completed | 11 (16.9%) |
| Discontinued | 41 (63.1%) |
| On-going | 13 (20.0%) |

cICI, consolidative immunotherapy; IQR, interquartile range; hypoCCRT, hypofractionated radiotherapy with concurrent chemotherapy; RWS, real-word study.

**Table S11. Adverse events during** **cICI period** **in the consolidation group of CA209-7AL (N=86), the RWS-cICI cohort (N=65), and the hypoCCRT-cICI cohort (N=65).**

| Adverse Event during cICI (N=65) | No. (%) | | |
| --- | --- | --- | --- |
|  | **Consolidation group of CA209-7AL (N=86)** | **RWS-cICI cohort (N=65)** | **HypoCCRT-cICI cohort (N=65)** |
| Led to discontinuation of cICI | 23 (26.7%) | 22 (33.8%) | 25 (38.5%) |
| Grade 3-4 | 9 (10.5%) | 10 (15.4%) | 9 (13.8%) |
| Dyspnea | 2 (2.3%) | 3 (4.6%) | 3 (4.6%) |
| Pneumonitis | 3 (3.5%) | 2 (3.1%) | 2 (3.1%) |
| Lymphopenia | 3 (3.5%) | 3 (4.6%) | 2 (3.1%) |
| Cough | 2 (2.3%) | 2 (3.1%) | 3 (4.6%) |
| Proximal bronchial  tree toxicity | 3 (3.5%) | 1 (1.5%) | 3 (4.6%) |
| Thrombocytopenia | 0 | 1 (1.5%) | 0 |
| Diarrhea | 0 | 1 (1.5%) | 1 (1.5%) |
| Hemoglobin  decreased | 0 | 1 (1.5%) | 1 (1.5%) |

Included were all grade 3~4 events. No Grade 5 adverse event was recorded. CCRT, concurrent chemoradiotherapy; RWS, real-world study.
